# Recent advances in ionic thermoelectric systems and theoretical modelling

**DOI:** 10.1039/d4sc04158e

**Published:** 2024-08-20

**Authors:** Nazish Jabeen, Muhammad Muddasar, Nicolás Menéndez, Mohammad Ali Nasiri, Clara M. Gómez, Maurice N. Collins, Rafael Muñoz-Espí, Andrés Cantarero, Mario Culebras

**Affiliations:** a Institute of Materials Science (ICMUV), Universitat de València PO Box 22085 E46071 Valencia Spain Mario.Culebras@uv.es; b Stokes Laboratories, School of Engineering, Bernal Institute, University of Limerick Limerick Ireland; c Institute of Molecular Science (ICMol), Universitat de València PO Box 22085 E46071 Valencia Spain

## Abstract

Converting waste heat from solar radiation and industrial processes into useable electricity remains a challenge due to limitations of traditional thermoelectrics. Ionic thermoelectric (i-TE) materials offer a compelling alternative to traditional thermoelectrics due to their excellent ionic thermopower, low thermal conductivity, and abundant material options. This review categorizes i-TE materials into thermally diffusive and thermogalvanic types, with an emphasis on the former due to its superior thermopower. This review also highlights the i-TE materials for creating ionic thermoelectric supercapacitors (ITESCs) that can generate significantly higher voltages from low-grade heat sources compared to conventional technologies. Additionally, it explores thermogalvanic cells and combined devices, discussing key optimization parameters and theoretical modeling approaches for maximizing material and device performance. Future directions aim to enhance i-TE material performance and address low energy density challenges for flexible and wearable applications. Herein, the cutting-edge of i-TE materials are comprehensively outlined, empowering researchers to develop next-generation waste heat harvesting technologies for a more sustainable future.

## Abstract 2

This review covers the recent advances of ionic thermoelectric (i-TE) materials which offer a compelling alternative to traditional thermoelectrics due to their excellent ionic thermopower, low thermal conductivity, and abundant material options.

## Graphical abstract

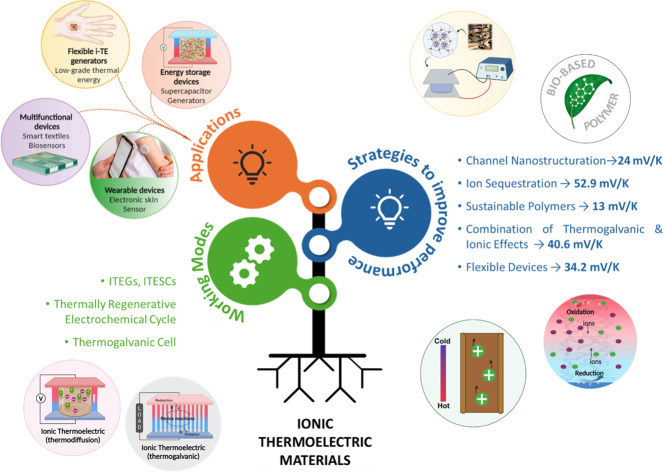

## 1. Introduction

Driven by the depletion of fossil fuels and the increasing frequency of climate disasters, the 21st century faces an unprecedented energy crisis. This has sparked significant interest from both academia and industry in exploring techniques for converting renewable power sources into electrical energy.^1^ Several technologies have been developed to harness heat, including thermoelectric generators (TEGs), thermoosmotic energy conversion, and organic Rankine cycles. One key area of exploration within TEGs involves leveraging the Seebeck effect of inorganic semiconductors (Bi_2_Te_3_), making them particularly effective in harvesting low-grade heat.^2–4^ The evaluation of their thermoelectric (TE) performance is commonly based on the dimensionless figure of merit (*ZT*), 
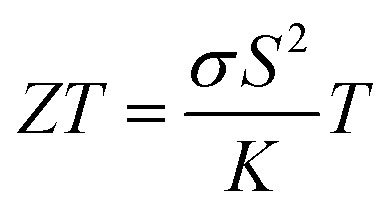
, where the incorporated factors are electrical conductivity (*σ*), the Seebeck coefficient (*S*), thermal conductivity (*K*), and absolute temperature (*T*).^5,6^

The literature reports the highest *ZT* at 3.1 at 783 K, and the highest *ZT* at room temperature at 1.3 approximately.^7,8^ However inorganic TE materials have drawbacks, including high costs, scarcity of elements, and limited mechanical flexibility. Efforts to enhance TEG performance are underway through nanostructuring existing materials, even though this approach comes with considerable production costs.^9^ On the other side, TEGs are gaining attention as a promising solution for powering wearable sensors and devices compared to heat engines. However, the mechanical stiffness of inorganic semiconductors and semimetals restricts their use in portable and wearable devices.^10^

Turning to electronic conducting polymers, their composition of abundant elements has sparked interest in recent studies for potentially lowering material costs.^11^ Despite substantial efforts and achievements in recent years, the optimized *ZT* of p-type organic thermoelectric materials still yields a relatively low Seebeck coefficient (maximum 70 μV K^−1^ for commonly produced polymers like PEDOT with a conductivity of 900 S cm^−1^). In contrast, the ratio of their electrical to thermal conductivities is already comparable to that of Bi_2_Te_3_ alloys.^12,13^ The interconnected material properties present a challenge in attaining a high *ZT* for electronic thermoelectric (e-TE) materials. Specifically, the Seebeck coefficient of e-TE is typically influenced by the transported excess energy or entropy to the Fermi level of charge carriers. Consequently, it tends to be considerably high in insulators characterized by large band gaps in their electronic band structures. However, the electrical conductivity is suppressed by such a wide bandgap. The e-TE materials with a Seebeck coefficient of ∼200 μV K^−1^ are typically optimized to achieve a high *ZT* or power factor, similar to the conventional Bi_2_Te_3_-based thermoelectric materials.^14,15^ Another difficulty in fabricating devices with such a low Seebeck coefficient is the large number of p- and n-type pairs required to achieve a reasonable voltage output, particularly in situations where there is a small temperature difference, as the case of harvesting thermal energy from the environment and the body to power small sensors. Consequently, there is a great need for new material classes and innovative ideas that can aid in resolving these issues.^15^

Recently, the discovery of “ionic thermpower” in electrolytes has introduced a novel phenomenon for heat-to-electricity conversion, which are hundreds of times larger than the electronic Seebeck coefficients found in conventional thermoelectric materials. It was established that the ionic Seebeck effect is the primary cause of the enormous thermopower, which can reach several mV K^−1^. An electrolyte that is subjected to a temperature gradient will undergo thermal diffusion of its internal ions until equilibrium is reached. The ions are finally reorganized, leading to the formation of an ion concentration gradient (also known as the Soret effect). This kind of ionic Seebeck effect can be found in gel electrolytes, inorganic solid electrolytes, ionic solids, and liquids.^16^ Among ionic thermoelectric (i-TE) materials, polymer-based electrolytes have gained attention due to their solid or gel states, facilitating easier device manufacturing. Additionally, i-TE materials possess excellent flexibility and stretchability, enhancing their potential for practical applications. These i-TE materials can be utilized in flexible TE devices to harness power from irregular heat sources.^17^ Two primary sources of ionic thermovoltage generation are Soret effect and thermogalvanic effect. Both effects involve an electrolyte for ion movement and electrodes for conducting currents and providing potential. The former effect is the thermodiffusion of ions that takes place in redox-free electrolytes. This phenomenon is primarily driven by the electric double layer (EDL), which forms as ions accumulate at the electrodes.^18^ However, the utilization of thermodiffusion among ionic charge carriers within an electrolyte has been implemented to convert heat into electrical energy, subsequently storing it in a supercapacitor.^19,20^ The later effect occurs in thermogalvanic cells based on thermogalvanic process. This effect arises from temperature-dependent entropy changes, originating from the electron transfer between redox molecules and the electrode.^16^

**Fig. 1:**
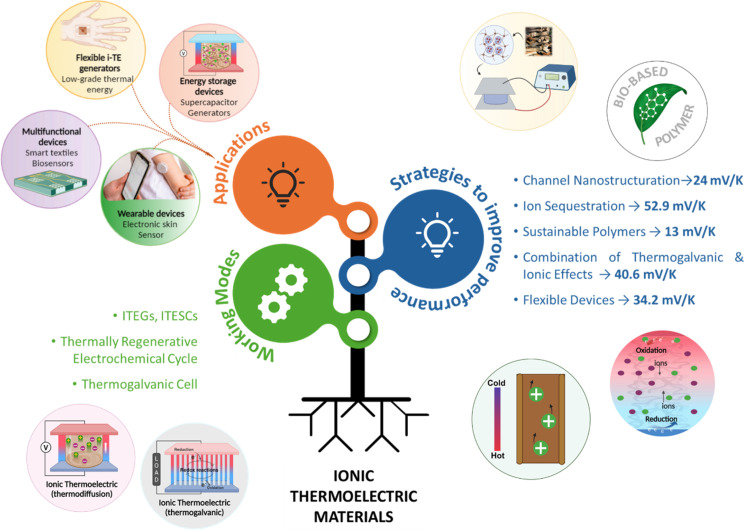
Recent advances in strategic development and application of ionic thermoelectric materials and devices.

Recent research on i-TE materials has made significant progress over the past decade as illustrated in Fig. 1. Shu *et al.* reported the fabrication of high-performance, flexible, and thermally stable carbon-based TE materials with an impressive power factor (*S*^2^*σ*) of 38.7 μW m^−1^ K^−2^. A fabricated TE device achieved a maximum power density of 79.3 nW cm^−2^ under a temperature difference of 20 K and a cold-side temperature of 300 K, showcasing the potential for cost-effective, high-performance TE applications.^21^ In the last decade, a novel i-TE film composed of carbon-based materials, an ionic liquid (1-butyl-3-methylimidazolium chloride, BMIM:Cl), and a crosslinker (*N*,*N*′-methylenebisacrylamide, MBA) was developed. The film exhibited enhanced TE performance, characterized by a high Seebeck coefficient (−76.7 mV K^−1^), power factor (753.0 μW m^−1^ K^−2^), and dimensionless figure of merit (*ZT*) of 0.19 at 383 K. A flexible, 9-legged thermoelectric device measuring 25 × 25 mm^2^ was fabricated, achieving a maximum power density of 1.32 mW cm^−2^ under a 20 K temperature difference. This work presents a promising foundation for developing high-performance, flexible i-TE materials and devices.^17^

Recent advancements in i-TE materials have shed light on their mechanisms and operational modes within ITESC materials by Zhao *et al.*^12^ 2017. They underscore the application of i-TE materials in energy conversion devices. Conversely, Wu *et al.* 2021 studies centered only on ionic conductors with promising applications in thermoelectric converters.^18^ Agar and their colleagues explored thermogalvanic cells, which utilize temperature-dependent redox pairs in the electrolyte to enhance the system's thermoelectric efficiency.^22^ In 2023 Shuai *et al.*^1^ review examined insightful strategies for thermo-electrochemical systems and TE performance. These reviews collectively elucidate the current state of research in the emerging field of ionic thermoelectrics covering i-TE mechanisms, material characteristics, figure of merit and energy conversion application. However, these reviews fall short of adequately highlighting the i-TE materials in energy integrated devices and ionic thermogalvanic cells based on electrochemical energy conversion system. Additionally, they do not provide a discussion on the utilization of combined ionic thermoelectric devices, as well as the significant categorization of theoretical modeling.^1,12,18,22^

This review will begin with a historical overview of i-TE materials, then move on to the Soret effect (thermodiffusion effect) and thermogalvanic effect, which serve as the primary driving forces behind the state of the art regarding the combined effect of both ionic systems involved in i-TE materials. The optimization parameters and theoretical models for TEG (one and two dimensional case) are outlined and examined in the final section. Consequently, the present review aims to not only stimulate the development of novel i-TE materials but also foster collaboration between researchers specializing in energy storage devices, thermodiffusion, and thermogalvanic phenomena. By bridging these areas of expertise, we can significantly advance our understanding of temperature-dependent ion transport and unlock applications beyond the capabilities of traditional electronic materials in low-grade energy harvesting technologies.

## 2. Historical overview of ionic thermoelectric materials

The exploration of ionic thermoelectric materials commenced with the investigation into ion thermodiffusion conducted by Swiss physicist and chemist Charles Soret (September 23, 1854, to April 4, 1904). Soret conducted experiments in which sodium chloride and potassium nitrate were placed in a vertical tube measuring 30 cm in length and 2 cm in diameter. The tube top and bottom ends were maintained at temperatures of 80 °C and room temperature, respectively. He observed that the concentration of salt solutions in the tube did not remain uniform. Between 1879 and 1884, Soret published several papers (originally in French) documenting his thermodiffusion experiments. These papers were later summarized and briefly discussed in English by Platten and Costesèque.^23^ The migration of ions induced by a temperature gradient resulted in the generation of a potential difference known as ionic thermopower. This phenomenon bears similarity to the Seebeck effect observed in electronic conductors or semiconductors, but the underlying principle differs significantly. Ionic thermopower is primarily governed by the disparity in ion contributions to conductivity and the transport numbers of cations and anions. Consequently, when there is a substantial difference in the sizes of anions and cations, high values of ionic thermopower are expected. One of the earlies works was carried out by Toshio Ikeda in the 60s measuring the ionic thermopower of the combination of ionic solutions based on: Ag, AgNO_3_; Cu, CuSO_4_; Ag, AgNO_3_; Cu, CuSO_4_; Hg, HgO, LiOH or NaOH or KOH; Hg, Hg_2_SO_4_, Na_2_SO_4_ varying the concentration from 0.01 to 2.0 molal.^24,25^ Moreover, the first studies of polyelectrolytes in particular, polystyrene sulfonic acid, were considered for thermocells during that decade.^26^ In latest 70s, starting to appear the first works utilising molten salts for ionic thermoelectric applications, in particular, mixtures of LiNO_3_ + AgNO_3_ and NaNO_3_ + AgNO_3_.^27^ However, it was not until 2016 when ionic thermoelectric materials started to be used as ionic thermoelectric supercapacitors with the work of Crispin *et al.*^28^ reporting the thermoelectric properties of a polymeric electrolyte based on polyethyleneoxide treated by NaOH with an exceptionally high ionic thermopower up to 11.1 mV K^−1^. During the last decade, research on ionic thermoelectric materials has been focused on hydrogels combining different electrolytes and natural based polymer matrices in particular cellulose and lignin. Highlights the work reported by Li *et al.*^29^ with a very high ionic thermopower up to 24 mV K^−1^ obtained for oxidized cellulose membranes. For the case of lignin, several works published by our group have been demonstrated the enormous potential of lignin-based membranes and hydrogels in combination with KOH as electrolyte as ionic thermoelectric materials reaching very high i*ZT* values (0.6–3.5).^6,30^ In addition, the combination of the ionic thermoelectric effect in thermogalvanic cells has recently opened avenues for maximising the thermoelectric performance. A good example of this is the work published in *Science* by Han *et al.*,^31^ where they developed wearable device consisting of 25 unipolar elements based on a gelatin matrix modulated with ion providers (KCl, NaCl, and KNO_3_) for thermodiffusion effect and a redox couple [Fe(CN)_6_^4−^/Fe(CN)_6_^3−^] for thermogalvanic effect. The device generated more than 2 volts and a peak power of 5 microwatts using body heat. In recent years, significant progress on thermoelectric fiber-based woven devices can be integrated with other functional components to create smart textiles. These components include moisture sensors, triboelectric generators, wearable current–voltage regulators, and energy storage units like tiny supercapacitors. Such integration of i-TE materials represents a valuable advancement for researchers working on flexible, wearable thermoelectric materials and devices, as well as their novel applications in customized thermal management and electronic skin.^32^

## 3. Ionic thermoelectric system

This section explores the essential principles underlying thermodiffusion and thermogalvanic systems, giving rise to the i-TE phenomenon. We explore several phenomenological models to understand how these effects result in the creation of an electric potential difference. The principles of heat transfer in i-TE materials are examined considering recent advancements.

### 3.1 Basic mechanisms

**Fig. 2:**
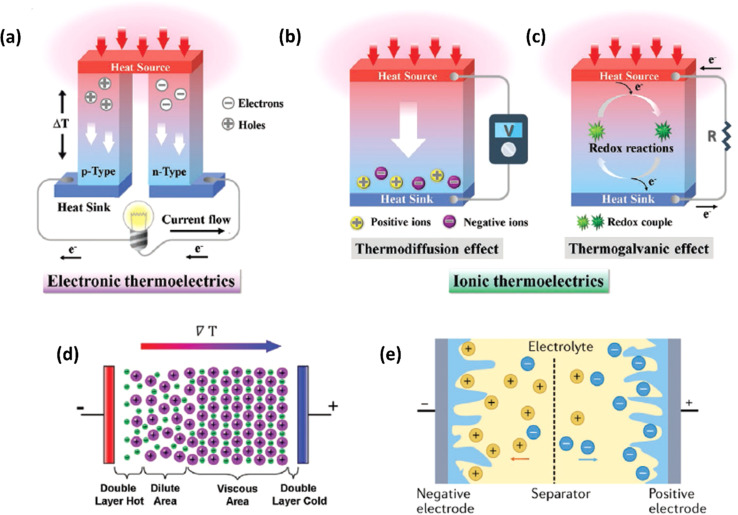
Representation of (a) diagram portraying a group of n-type and p-type e-TE materials, (b) thermodiffusive-based i-TE materials, (c) thermogalvanic-based i-TE material, (d) the internal structure of a thermodiffusive-based i-TE materials in its operational mode, reproduced with permission.^1^ Copyright 2023, Wiley-VCH. (e) Electric double-layer in supercapacitor, reproduced with permission.^33^ Copyright 2019, Elsevier.

Electronic (e-TE) and ionic thermoelectric (i-TE) materials both offer pathways for converting heat into electricity, but their underlying mechanisms differ significantly. In e-TE materials (Fig. 2a), the Seebeck effect drives the process. Under a temperature gradient (Δ*T*), electrons (or holes) act as charge carriers, propelled by an electromotive force.^34^ Due to their negative charge, electrons migrate from the hot to cold side, generating an electric potential difference against the temperature gradient. i-TE materials, however, rely on two distinct sources for potential generation: thermodiffusion and the thermogalvanic effect. Thermodiffusion leads to ion concentration gradients within the material due to Δ*T*, creating an internal potential. Additionally, the thermogalvanic effect harnesses the temperature dependence of electrode/electrolyte interactions, resulting in another potential contribution, as depicted in Fig. 2b and c.^1^

### 3.2 Thermodiffusion based system

The thermodiffusion-based system, originating from the Soret effect that was first discovered by Soret and Ludwig in the 19th century, plays a pivotal role in i-TE materials. Under a Δ*T* ions migrate from the hot to cold side, leading to concentration imbalances at the electrodes. To maintain electrical neutrality, both cations and anions participate in this process within the i-TE material. However, the distinctive migration speeds (diffusion rates) of these ions are influenced by variations in size, coulomb force and activation energy, resulting in different diffusion rates between cationic and anionic ions. This leads to an imbalance in the concentrations of each type of ion. As shown in Fig. 2d, a double-layer region containing a Helmholtz layer and a diffusion layer form close to the electrolyte/electrode interface following the Gouy–Chapman–Stern theory.^35^ The difference in ion concentration causes a dilute region to be near one electrode and a viscous region near the other, which results in different chemical potentials in each electrode's Helmholtz layer. Thus, an electric potential difference between the two electrodes is initiated by the chemical potential difference between the two Helmholtz layers.^18^

A recent study on i-TE materials, inspired by Soret effect, draws a parallel intention with e-TE materials based on the Seebeck effect, as both exhibit the ability to generate thermovoltage associated with charge transfer enthalpy. However, some noteworthy differences are present. (i) Ions can have charges significantly higher than electrons, depending on their overall valence state. Electrons have a charge of one unit. (ii) Unlike ion transport, electron transport is confined within a particular energy window and follows the Fermi–Dirac statistical functional. (iii) Electrons are the only carriers that contribute to electrical conductance in e-TE materials, while cations and anions are also involved in i-TE materials. Importantly, (iv) electrons can traverse an external circuit, while ions accumulate at electrodes, leading to the formation of an electric double layer (EDL), as depicted in Fig. 2e. This process induces a transient thermoinduced current, illustrating the charge stored in the EDL capacitors. By utilizing high-capacitance electrode materials, this accumulated charge can be significantly increased, enabling the charging of a supercapacitor or a battery.^1^ This principle is employed in the design of ionic thermoelectric supercapacitors (ITESCs), which combine the functionalities of a thermoelectric generator and a supercapacitor. The establishment of an equivalent circuit, combining a TEG and a supercapacitor, is crucial for electronic material-based thermoelectric applications.^36,37^

### 3.3 Ionic thermoelectric generators

**Fig. 3:**
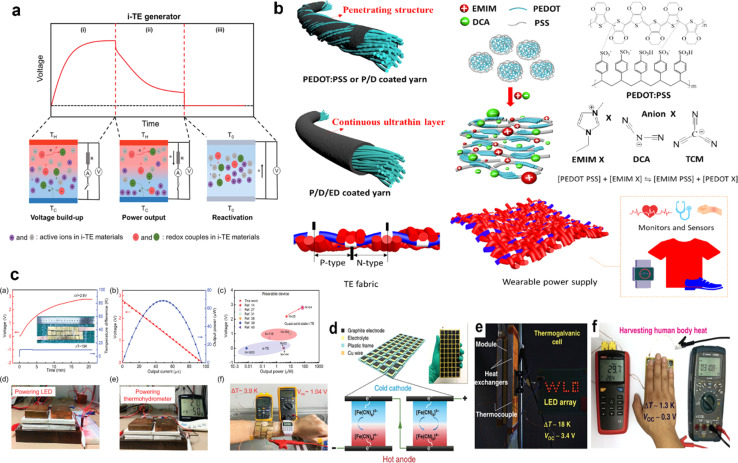
(a) Schematic representation of ITEGs, reproduced with permission.^39^ Copyright 2022, Wiley-VCH. (b) Chemical interactions between PEDOT:PSS and [EMIM:DCA], reproduced with permission.^1^ Copyright 2023, Wiley-VCH (c) voltage output of a wearable i-TE device, reproduced with permission.^39^ Copyright 2022, Wiley-VCH. (d–f) Photographs of the i-TE generators, reproduced with permission.^1^ Copyright 2023, Wiley-VCH.

Ionic thermoelectric generators (ITEGs) are gaining recognition for their ability to efficiently recover low-grade waste heat. However, their practical application is hindered by low power and energy density. To address this, scientists have developed innovative ITEGs with enhanced power and energy density, enabling continuous charging of small devices. By synergistically combining electrode redox reactions with the thermodiffusion effect, these ITEGs demonstrate impressive thermoelectric conversion properties. This technology holds commercial promise for powering wearable electronics and sensors, opening new avenues for advanced ionic thermoelectric devices with improved heat-to-electricity conversion and storage capabilities.^38^ Li *et al.*^29^ introduced a generator mode where an i-TE cell is directly connected to a load resistance after voltage build-up. This study reported the three stages ITEG mode as illustrated in Fig. 3a. Voltage build-up: under a temperature difference (Δ*T*), a voltage is generated across the i-TE material due to the movement of ions from the hot to the cold side. Power output: once a sufficient voltage is built up, an external circuit can be connected to the i-TE device. This allows for the flow of ions and the generation of electrical power. Reactivation: to maintain consistent power output, the i-TE device needs to be reactivated. This involves removing the temperature difference (Δ*T*) to allow for the redistribution of ions within the material, preparing it for the next charging and discharging cycle.^39^

Fig. 3b demonstrate a i-TE generator fabricated by coating a fabric with a PEDOT/DMSO/[EMIM] solution.^40^ This process yielded a continuous, 8.78 μm-thick TE layer on the cotton yarn, contrasting with the discontinuous structure of pristine PEDOT-coated yarn. This enhancement is attributed to the PEDOT/DMSO/[EMIM] composite's higher viscosity and surface tension, preventing yarn penetration. The surface-coated yarn demonstrated significantly improved flexibility compared to its pristine counterpart. Moreover, the maximum power factor (*S*^2^*σ*) was optimized to 24.7 μW m^−1^ K^−2^, a substantial improvement over the pristine PEDOT value of 0.00684 μW m^−1^ K^−2^. Researchers have focused on practical application of long-term power generation of the gelatin–KCl–FeCN^4−/3−^ i-TE cell by designing 3D hierarchical Au/Cu electrodes as shown in Fig. 3c. The optimized i-TE cell shows high output power and thermopower, making it a potential electricity source for sensors and wearable electronics. This watch-strap-styled device generates 2.8 V and 68 μW when worn on the arm, with a skin temperature of 30 °C and an environmental temperature of 20 °C, resulting in a Δ*T* of approximately 10 K. The high output voltage can drive sensors without additional DC–DC voltage boosters, as most sensors operate within a voltage range of 1.5–3.6 V. Compared to other i-TE and electronic thermoelectric (e-TE) devices, optimized devices demonstrated the output voltage and power are significantly 127% and 1360% respectively, and substantially higher than other reported devices. The device can directly power various electronics, including an LED bulb and a thermohydrometer. It also harvests heat from the body, generating a stable voltage of approximately 1.04 V under a small Δ*T* of approximately 3.9 K in an indoor summer environment, enough to drive a digital watch without voltage boosters.^39^ Additionally, a thermogalvanic i-TE module was developed using guanidine chloride (GdmCl) and urea-enhanced [Fe(CN)_6_^4−^/Fe(CN)_6_^3−^] electrolytes. A polyamide frame housed 50 individual units, each containing a graphite electrode sandwiching the electrolyte. This module generated an open-circuit voltage of 3.4 V and a short-circuit current of 1.2 mA under an 18 K temperature difference, capable of powering an LED array. Notably, the device effectively harvested human body heat, producing a stable voltage of over 0.3 V under a minimal temperature difference of 1.3 K^41^ (Fig. 3d–f).

### 3.4 Ionic thermoelectric supercapacitors

**Fig. 4:**
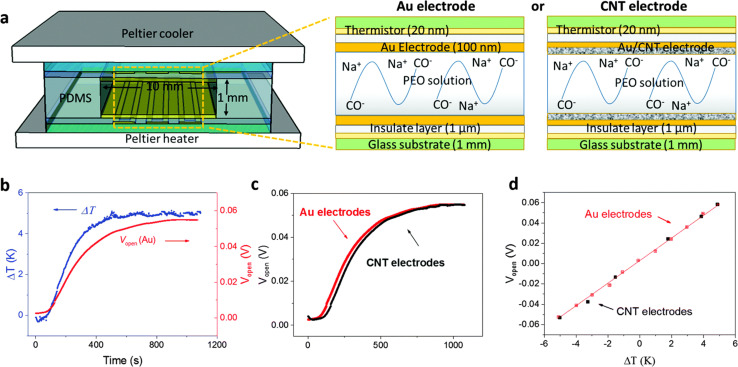
(a) Illustration of the ITESCs device (left) featuring two distinct electrodes (right, Au and CNT), along with the reaction occurring in the solution. (b) Recorded *V*_thermo_ and Δ*T* during the heating process using the Au electrode. (c) The observed *V*_thermo_ while heating with either CNT or Au electrodes. (d) *V*_thermo_ at various Δ*T* values using CNT electrodes (black solid squares) and Au electrodes (red open squares), reproduced with permission.^28^ Copyright 2016, Royal Society of Chemistry.

Over the last few years researchers reported that ionic thermoelectric supercapacitors (ITESCs) create a transient thermo-induced current by forming an EDL at the electrodes through the aggregation of thermally diffused ions. Supercapacitors or batteries can be charged by using high-capacitance electrode materials, which can greatly increase the accumulated charge.^42^ Zhao *et al.* explored the thermoelectric characteristics of the PEO-NaOH solution by injecting the liquid electrolyte contained within a 1 mm thick, 10 mm diameter cylindrical chamber. The planner surfaces of Au electrodes were deposited on each side, making direct contact with the polymer electrolyte (see Fig. 4a). Thermistors embedded beneath the electrodes monitored the temperature difference (DT). The open voltage induced by Soret under different Δ*T* conditions was explored while subjecting one side of the device to heating and the other side to cooling, as depicted in Fig. 4b. The investigation of thermovoltage was conducted within various Δ*T* conditions between 25 to 35 °C. Five minutes after stabilization at each Δ*T*, the voltage was recorded. The outcomes, showcased in Fig. 4c, reveal a linear variation of *V*_thermo_ with Δ*T*. From the linear fit, the researchers calculated an ionic Seebeck coefficient of +11.1 mV K^−1^ for the PEO-NaOH electrolyte. Using both CNT and Au electrodes yields similar saturation of *V*_thermo_*vs.* time (Fig. 4c) and the evolution of *V*_thermo_*vs.* Δ*T* (Fig. 4d). This suggests that electrodes nature does not influence the results, indicating that the thermovoltage is the inherent property of the polymer electrolyte.^28^ However, in recent years, researchers have found that the thermopower of ionic thermoelectric devices does not solely depend on the intrinsic properties of the materials but also includes a significant contribution from electrode polarization, necessitating careful consideration and correction in measurements.^43^ This can be expressed as Δ*V* = *S*Δ*T* + Δ*V*_electrode_, where Δ*V* is the total measured voltage difference, *S*Δ*T* is the thermoelectric voltage, and Δ*V*_electrode_ is the voltage contribution from electrode polarization. When an inert metal is used as electrode, the term of Δ*V*_electrode_ could be negligible.

### 3.5 Operational mechanism of ITESCs

**Fig. 5:**
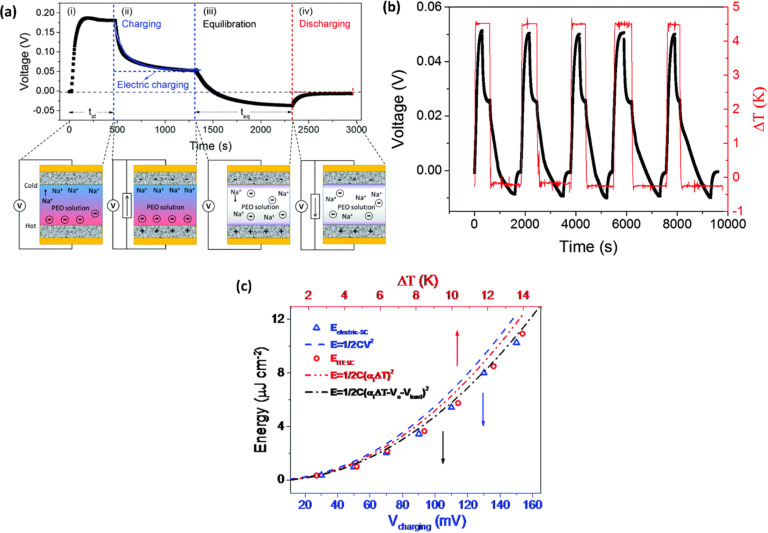
Operation principle of ITESC (a) schematic mechanism: (i) Δ*T* generates ionic thermal voltage, (ii) thermoelectric charging, (iii) Δ*T* removal for ion equilibrium, (iv) discharging. (b) Charging/discharging with periodic heating, reproduced with permission.^44^ Copyright 2017, Wiley-VCH. (c) Depicts the energy density of the ITESC compared to both Δ*T* and electric charging methods. The red open circles represent the data for the ITESC, while the blue open squares represent electric charging. The dashed lines show theoretical predictions based on thermal voltage (*V*_thermo_, dashed red line), electric voltage (*V*_electric_, dashed blue line), and effective voltage (*V*_effective_, dashed and dotted black line), reproduced with permission.^28^ Copyright 2016, Royal Society of Chemistry.

Wang *et al.*^44^ reported the four distinct steps that comprise the comprehensive operational mechanism of ITESC in Fig. 5a, making it easier to extract energy from i-TE materials. The first step of the charging process described, setting up the device for a Δ*T*, which causes an open circuit to produce thermal voltage.^45^ The representation in Fig. 5a has a positive thermal voltage arise at the cold side, primarily due to dominant sodium cation thermodiffusion. Moving on to the second step, the EDLC is charged by connecting the electrodes, *via* a load or a short circuit. In this stage, an electric current flow from the electrodes with a higher electric potential to the one with a lower potential, all while maintaining the Δ*T*. The third step involves an equilibration period, involves removing the load and the Δ*T* from the circuit. Importantly, the charges that were stored at the electrode–electrolyte interface did not disappear even as the ions diffused back. Since there is no thermal voltage generated, stored charge at electrodes controls the ensuing open circuit voltage at Δ*T* = 0, which should be observed in this step with a sign opposite to initial thermal voltage in step I. Finally, the fourth step involved the loss of stored charge due to the EDLC discharging through an external circuit load resistance. Once the charging process is finished, the DT is no longer necessary and is only needed for stages I and II. After completing a full cycle, brings the ITESC back to its initial state.^12,44^ The repeated cycling yields highly reproducible charge–discharge current patterns, as illustrated in Fig. 5b. These observations underscore the stability of the device and suitability for harvesting energy from fluctuating heat sources. This characteristic also makes it possible to store and use energy without a constant heat source. Such a device is particularly well-suited for use with intermittent heat sources like solar energy, allowing capacitors to charge during the day and discharge during the night.^28^

### 3.6 Stored charge and energy

To understand the charging mechanism of the ITESC, Dan Zhao *et al.* investigated the relationship between the stored electrical energy in the supercapacitor and the ionic thermopower, signifying the thermal voltage generated by the electrolyte. This involved charging and discharging the ITESC under different Δ*T* and subsequently calculating the energy stored in the supercapacitor by integrating the current over time across a load resistance.^12^1
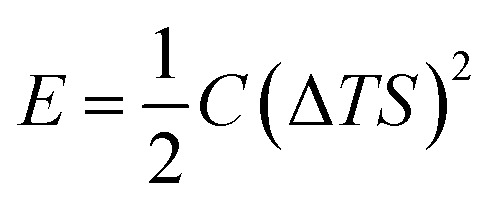
where *C* is the capacitance, *S* is ionic thermopower and Δ*T* is applied temperature gradient. Fig. 5c illustrates Dan Zhao *et al.* findings, that the measured stored energy (depicted as red dots) exhibits a quadratic increase in relation to the Δ*T* (indicated by the red dashed line). Additionally, they plotted the stored energy obtained in the device when charged by an external power supply, against the charging potential (Fig. 5c by the blue line and triangles) and overlaid it with the thermally charged curve. Their investigation demonstrated that the increase in both the generated and stored electric energy follows a quadratic relationship with the ionic thermopower. The relationship was directly linked to the charging potential, as expressed by the equation *V* = Δ*T*·*S*.^12,42^

### 3.7 ITESCs for integrated energy conversion-storage

**Fig. 6:**
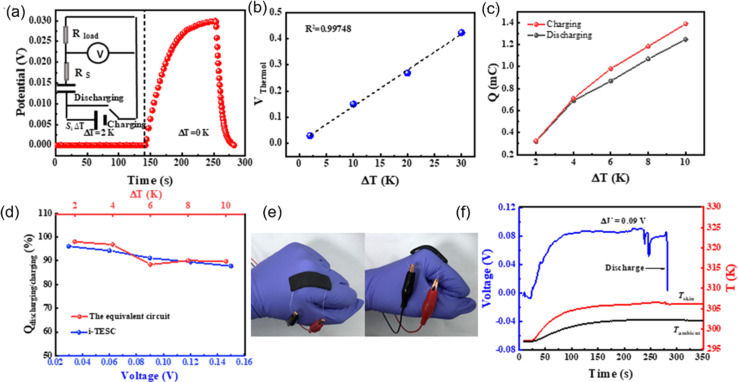
Representation of (a) equivalent circuit and charge–discharge characteristics of ITESCs. (b) Thermal voltage of ITESCs *vs.* Δ*T*. (c) Stored charge as a function of Δ*T*. (d) The charge–discharge ratio in relation to both Δ*T* and voltage. (e) Potential applications of ITESCs in wearable electronics. (f) Output voltage from a prototype stretchable ITESC worn on the back of a hand, reproduced with permission.^46^ Copyright 2022, Wiley.

Drawing inspiration from the work of Xinyu Yang *et al.*,^46^ they proposed a model for understanding the charge–discharge behavior of an ITESC based on an equivalent circuit (as shown in Fig. 6a). Within this framework, the thermal voltage arising from the DT acts as the driving force. During stages i and ii, the absence of voltage signals points towards an equilibrium state within the device, with electrons and holes balanced at the positive and negative electrodes.^47^ It is crucial to note that a temperature gradient of 0 K would result in minimal redistribution of Na^+^ ions within the electrolyte interface. Additionally, without a counterbalancing charge attraction, electron and hole movement across the interface would not be expected. When the external circuit remained disconnected during stages i and ii, limited ion transport resulted in an inherent charge imbalance. This imbalance generated a potential difference across the positive and negative electrodes, measurable as the open-circuit voltage (Fig. 6b). Analysis revealed a linear relationship between this voltage and the applied DT, with the highest recorded voltage of 0.45 V achieved at ∼30 K DT. Further investigation conducted a series of calculations to evaluate the quantity of charge transferred during stage iii. The results presented in Fig. 6c illustrate a nearly linear correlation between the amount of charge and the loop, contingent on the DT. However, discharge capacity suffered a notable decline due to internal resistance contributing to a voltage drop. Xinyu Yang reported a more comprehensive understanding of the energy consumption dynamics during both charging and discharging processes, to assess the charge–discharge ratio in relation to Δ*T* and voltage. Fig. 6d showcases the remarkable consistency of the charge–discharge ratio across varying DT and initial voltage conditions. Notably, at 50 mV (*T* = 2 K), the ratio remains near 96%, highlighting the substantial charge storage capacity and exceptional stability of the ITESC. Further investigation explored the device's tensile stability by subjecting it to 20 cycles of 20% stretching. Subsequent charge–discharge analysis revealed a consistent linear relationship between voltage and the ratio across a 30–150 mV range, regardless of pre- or post-stretching conditions. This finding underscores the notable tensile stability of the integrated device. Xinyu Yang. *et al.* reported the practicality of integrated i-TE device into wearable electronics. As shown in Fig. 6e, a flexible and stretchable device was applied to the back of the hand, simulating real-world interactions with the human body. The device effectively harvested low-grade heat during palm bending, generating a voltage output of 90 mV at a temperature difference of 6 K (Fig. 6f). This finding highlights the applicability of such devices in powering wearable electronics.^46,48^

### 3.8 Ionic thermoelectric figure of merit

Over the past three years the researcher successfully explained that ionic figure of merit (i*ZT* = *σ*_*i*_*S*_*i*_^2^*T*/*K*), served as a key parameter for assessing the performance of i-TE materials, drawing parallels with their electronic counterparts. It is crucial to highlight that i*ZT* for electrolytes does not exhibit the same correlation with heat to electron conversion efficiency as observed in traditional thermoelectric materials, mainly due to the limitations in utilizing ionic materials in conventional TEGs.^12^ Zhao and their colleagues described a comprehensive study into the figure of merit for ITESC.^12^ The assessment of charging performance involved determining the ratio between stored electric energy and absorbed heat during the charging process, with expression detailed in eqn (2). Using this equation,2
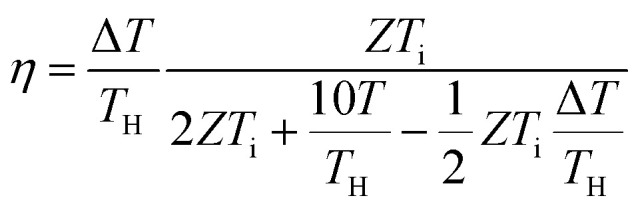


It is important to mention here that this formula is applicable only to ITESC. Currently, there is a lack of a universal ionic thermoelectric *Z* that can serve as a comprehensive figure of merit for all the ionic thermoelectric systems. A recent study by Ma *et al.* have introduced a new figure of merit for the thermodiffusion based i-TE materials (eqn (2a)).^49^2a
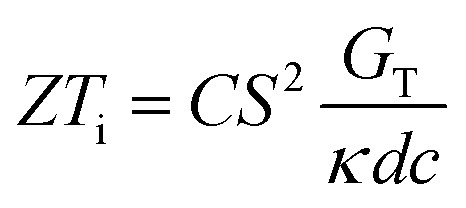


Previous work introduced novel parameters to characterize thermodiffusion-based ionic thermoelectric (i-TE) devices, including capacitance (*C*), thermopower (*S*), and a factor (*G*_T_/(*κdc*)) representing the relaxation of temperature difference. This research offered valuable insights into device operation but overlooked the influence of ionic conductivity and the discharge process. Consequently, a more comprehensive figure of merit is necessary to fully understand i-TE device performance. Recent studies by X. Qian, R. G. Yang, and colleagues have addressed these limitations by incorporating ion transport dynamics and electrochemical reactions into the figure of merit (*Z*) for i-TE devices that leverage both thermodiffusion (TD) and thermogalvanic (TG) effects.^50^2b
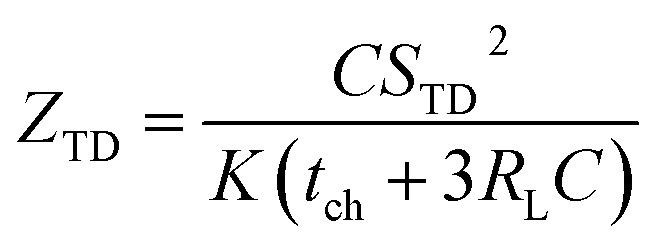
2c
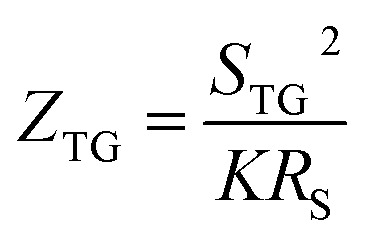
where, *K* is the thermal conductance. *t*_ch_ is the thermal charging time of i-TE supercapacitor mode. *R*_L_ is the external load resistance of i-TE supercapacitor mode. *R*_s_ is the overall resistance of i-TE cell of i-TE generator mode.

They considered a critical parameter *t*_ch_ when i-TE devices work. However, combining these above two equations is still a big challenge. This gap in research hinders our ability to accurately compare and optimize various ionic thermoelectric systems, as current figures of merit often do not account for the unique interplay between thermogalvanic and thermodiffusion effects.

**Fig. 7:**
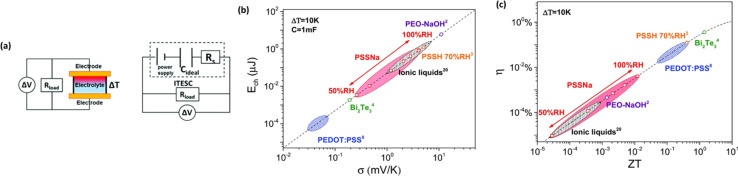
A comparison is presented between ionic and electronic thermoelectric materials within an ITESC. (a) Displays the experimental setup for ITESC measurement (left) and the corresponding equivalent circuit, highlighting the setup for experimental measurements (right). (b) Efficiency comparisons are drawn between different materials within the ITESC based on their *ZT*. (c) The impact of material choice on the ITESCs stored energy capacity by varying *ZT* values, reproduced with permission.^44^ Copyright 2017, Wiley-VCH.

The investigation of research team described that effectiveness of an electrolyte, denoted by i*ZT*, plays a potential role in determining the efficiency of an ITESC employing this specific material. A direct comparison between ionic and electronic thermoelectric materials proves challenging due to the distinct operational characteristics of a TEG and an ITESC. However, a meaningful comparison can be drawn by examining ionic materials within an ITESC featuring capacitance and electronic materials within a TEG coupled in series with SC of capacitance (*C*). This comparison is justified as they share analogous equivalent circuits, as shown in Fig. 7a. Consequently, a unified graph can depict the energy conversion efficiency of both electronic and i-TE materials by considering ITESCs as equivalent to series-connected TEG-SC circuit (as illustrated in Fig. 7b), this approach has limitation. In practical applications the efficiency of converting heat to stored electricity in an ITESC using electrolytes is still lower compared to Bi_2_Te_3_ in a TEG-SC circuit, mainly because of the significantly lower ionic conductivity. Conversely, as presented in Fig. 7c, electrolytes facilitate the storage of significantly larger electrical energy per leg/per Δ*T*. This phenomenon is attributed to the square of the ionic thermopower (as per eqn (1)), that is substantially higher for polymer electrolytes (∼10 000 μV K^−1^) compared to Seebeck coefficient of Bi_2_Te_3_ alloys (∼200 μV K^−1^). Future research should focus on simultaneously improving ionic conductivity and maintaining a high ionic thermopower in materials designed for ITE applications. Interestingly, devices utilizing either a series circuit where a TEG charges a SC or a single ITESC system can offer pulsed electrical power through the discharge of the SC, addressing the limitation of continuous low power output encountered with TEGs alone.^6^ However, beyond material advances, unlocking the full potential of i-TE for practical applications introduces additional challenges related to device design. The continuous power generation capability of ionic thermoelectric devices encounters a fundamental challenge due to the necessity for discharging in the absence of a temperature difference. Ionic thermoelectric concepts demonstrate particular effectiveness in converting intermittent heating/cooling scenarios. Through deliberate design considerations, the charging duration of the supercapacitor can be synchronized with the source of heat fluctuation cycle. This presents opportunities for integrating with other steady-state power generation systems like TEGs and solar cells, both reliant on heating or illumination for operation. However, it is crucial to address the inherent limitation of low energy density in i-TE devices.^51^ However, this challenge is amenable to resolution through circuit manipulation. For example, harnessing the ITESC to charge capacitors directly provides a means to mitigate this concern. Additionally, adjusting the circuit configuration from parallel to series during energy utilization offers a pathway for enhancing energy density. This strategic manipulation of the circuit holds promise for overcoming the limitations associated with the energy devices of i-TE devices.^44^

### 3.9 Hydrogels in ITESC for thermoelectric energy systems

**Fig. 8:**
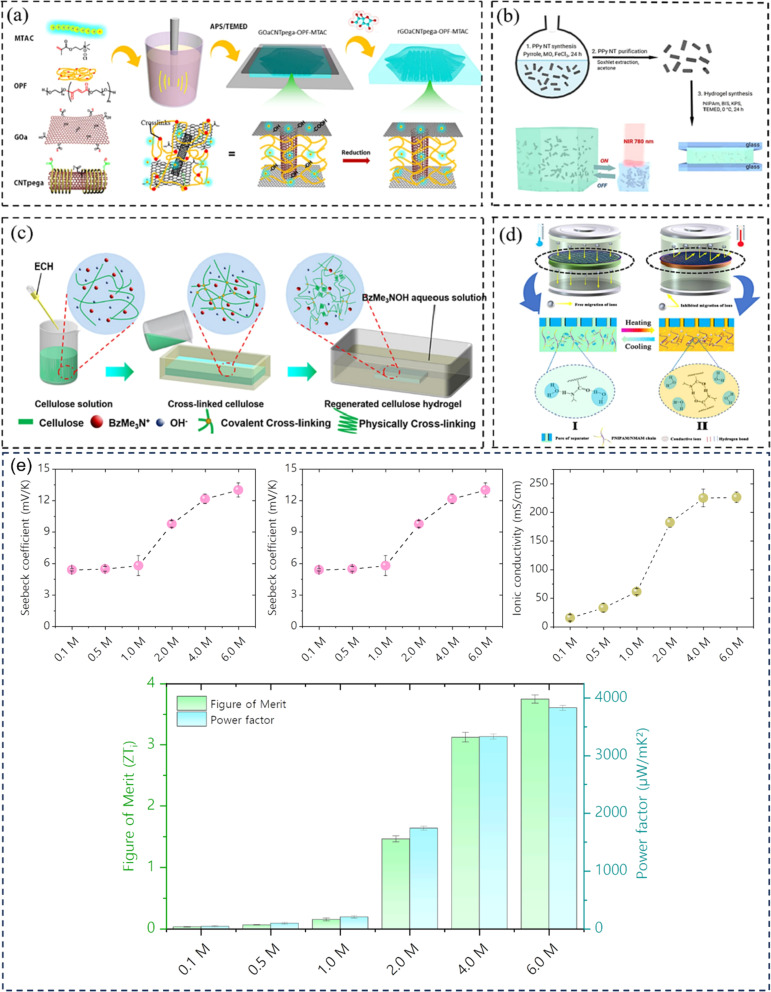
(a) Preparation for a conductive composite hydrogel (GO_a_CNT_pega_-OPF-MTAC), reproduced with permission.^54^ Copyright 2017, American Chemical Society. (b) Reversible on-demand adjustment of ITESC capacity through light changes. (c) Synthesis of a cellulose-based ionic conductive hydrogel using entangled chain networks. (d) The schematic representation of PNIAPM-*co*-NMAM polyelectrolytes highlighting their self-protective and thermal-switching capabilities in energy storage devices, reproduced with permission.^53^ Copyright 2023, Elsevier. (e) Ionic thermoelectric properties of TcB9-2.5% lignin-based hydrogel infiltered with KOH electrolyte, reproduced with permission.^6^ Copyright 2024, Springer.

Hydrogel can boost thermal capacity, make TE material more flexible, and stop leakage of electrolyte.^52^ In order to enhance thermoelectric conversion, it can help electrodes or act as an electrolyte in ITECS. In the hydrogel ITESC principle, an active substance with capacitive characteristics serves as electrode, and a TE hydrogel based on the Soret effect behaves as electrolyte. A potential difference is created at each end of an ionic electrolyte because of concentration difference due to thermal diffusion of carriers in the presence of Δ*T*. Ions build up at the electrode surface transition as a result of the incapacity of ion transfer and electron between electrolyte and electrode. At this point, when both electrodes are activated, they create an induced current, as a result EDLC forms between electrolyte and electrode, and the electrode stores the converted electrical energy.^53^ When applied to the ITESC electrode, i-TE hydrogel offers numerous benefits. The rich and uniform porous structure facilitates ions movement from electrolyte to reach electrode surface as well as to increase the electrode material's specific surface area and contact area when the polymer is dispersed around the electrolyte and pores. The electrode surface can retain more charges for high specific surface area capacitance during the formation EDLC. Liu *et al.* in 2017 reported smart responsive hydrogels, such as poly(*N*-isopropylacrylamide) (PNIPAM), are uniformly and chemically dispersed on material of electrode (Fig. 8a).^54^ Elashnikov *et al.* in 2021 demonstrated that these hydrogels can be used for external transport and charge storage control, allowing ITESC's electrical capacity to be adjusted on-demand (Fig. 8b). They can also control the disruption or formation of the electrode material's conductive network by changes in light or temperature. The stability of ITESC can be affected by repeated charging and discharging of hydrogel materials, which are highly dependent on their water content. This can be mitigated by encapsulating the hydrogel with the use of cast stretchable polymer or impregnated coatings, or by weakening the interactions between the hydrogel and water by adding double network structures, hydrophobic linkages, ionic complexes, *etc.*^55^ On adding hydrogels to ITESC electrolytes, efficient ion-selective networks are formed by entangled molecular chains. In addition, good processability of electrolytes in hydrogel offers additional flexibility in ITESC process design, such as area, thickness *etc.*, which facilitates the easy fabrication of ITESCs are reported by Horike *et al.*^56^ in 2020. However, at a specific Δ*T*, the migration of anions and cations in pure hydrogels is hindered by the electrostatic interactions produced by entangled chains. These interactions close the hydrophobic transition and ion-conducting channels on the surface of hydrogel, lowering electrical conductivity and influencing thermoelectric conversion as show in Fig. 8c.^57^ Additional approaches include building crosslinked networks with cation selectivity or increasing the concentration gradient between the cations and anions. When the hydrogel electrolyte is a low critical solution temperature responsive polymer, such as PNIPAM, methyl cellulose (MC) and pluronic[poly(ethylene oxide)-*block*-poly(propylene oxide)-*block*-poly(ethylene oxide) (PEO–PPO–PEO)], reversible thermal switching self-protection of ITESC is also possible. Zhang and colleagues, for instance, created poly(*N*-isopropylacrylamide-*co-N*-methylolacrylamide) (PNIPAM/NMAM) hydrogel polyelectrolytes. As demonstrated in Fig. 8d, a mechanism of self-protection was achieved in hydrophobic segment of PNIPAM through thermally induced switching. Though this is a feasible approach, there has not been much research done on ion-conductive hydrogel electrodes particularly for ITESC when both electrode and electrolyte materials are made up of hydrogel.^53,58^

To address these drawbacks, one of studies reported the synthesis of lignin-based hydrogels, with improved thermoelectric properties. This study demonstrates the effect of adjusting electrolyte concentration within the hydrogels. Notably, TcB9-2.5% hydrogels infiltrated with 6 M KOH electrolyte exhibit a compelling combination of properties for ITESC applications. As shown in Fig. 8e, these hydrogels achieve a high ionic conductivity 226.5 mS cm^−1^, a remarkably low thermal conductivity 0.29 W m^−1^ K^−1^, and an impressive ionic thermopower of 13 mV K^−1^, resulting in a superior 3.75 ionic figure of merit (i*ZT*).^6^ These exceptional i-TE characteristics are further enhanced by the biocompatibility, environmental friendliness, and biodegradability of lignin-based hydrogels. This unique combination opens doors for their utilization in a wide range of high-value energy applications. Promising areas of implementation include environmental temperature sensors, healthcare-oriented biomedical sensors, and wearable electronics engineered for efficient and sustainable energy harvesting. This development not only represents a significant advancement in the field of ionic thermoelectricity but also holds immense potential for both promoting environmental sustainability and fostering technological progress.

## 4. Thermogalvanic cells

**Fig. 9:**
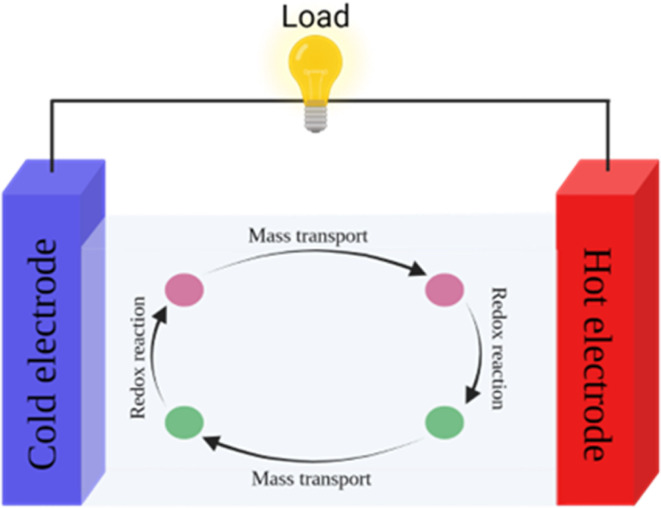
Schematic diagram of a TGC's operation.

Thermogalvanic cells (TGCs) are a type of ionic thermoelectric materials consisting of electrodes surrounded by an electrolyte where the charge carriers are the ions contained in the electrolytes of the cells. TGCs exploit electrochemical processes within liquid, solid, or quasi-solid electrolytes to convert temperature differences into electrical potential.^59^ These systems offer similar advantages to other ionic thermoelectric materials when compared to traditional thermoelectric materials such as the higher thermopower and the compatibility with various temperature ranges, which positions them as promising avenues for energy harvesting.^60^ This section delves into the fundamental principles and recent advancements of thermogalvanic systems within the broader context of ionic thermoelectric materials. We will explore the underlying electrochemical mechanisms, the role of electrolytes, and key factors influencing the performance of these systems. Understanding the intricacies of thermogalvanic cells is essential for harnessing their full potential and realizing their role in the transition towards more efficient and sustainable energy conversion technologies. At the heart of thermogalvanic systems lies the principle of electrochemical conversion of thermal energy. As previously mentioned, these systems typically consist of two identical electrodes and an electrolyte; when exposed to a temperature gradient across the cell, electrochemical reactions occur at the electrode–electrolyte interfaces.^61^ The temperature difference induces unequal chemical equilibriums on either electrode, generating an electric potential difference which can be harnessed to produce electrical power through the flow of electrons along the outer circuit. A schematic representation of a TGCs working mechanism is illustrated in Fig. 9. Importantly, the key to efficient operation in thermogalvanic systems is the appropriate selection of electrode materials and electrolytes that enable optimal ion concentration, electrolyte diffusion, charge transfer resistance at the electrode/electrolyte interface, and electrical conductivity of the electrodes.^62,63^

As has been described, TGCs operate in a similar manner than other ionic thermoelectric materials, and as such, have a ionic thermopower or ionic Seebeck coefficient, or thermopower, equivalent of their own which is defined in eqn (3) as:^64^3
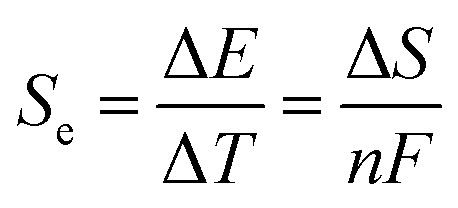
In the given equation, Δ*E* represents the open-circuit voltage, Δ*T* denotes the temperature difference, *n* stands for the number of electrons transferred in the redox reaction, *F* is Faraday's constant, and Δ*S* represents the partial molar entropy difference of the redox couple.

Another parameter of interest for TGCs is the Carnot-relative efficiency, which denotes the theoretical maximum of energy conversion efficiency that the TGC can achieve, shown in eqn (4):^65,66^4
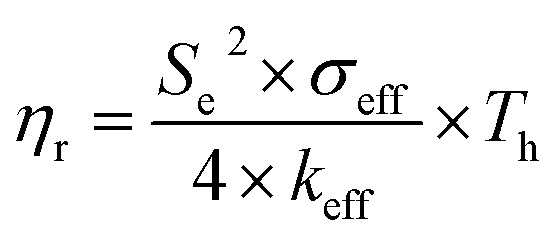
where *σ*_eff_, *k*_eff_ and *T*_h_ represent the effective electrical conductivity, the effective thermal conductivity, and the temperature at the hot side of the TGC, respectively. It is important to note that the Carnot-relative efficiency is temperature-dependent, and as such test conditions need to be known to use it to compare the performance of different thermocells. As of now, the ferri/ferrocyanide (Fe(CN)_6_^3−^/Fe(CN)_6_^4−^) couple stands out as one of the most effective redox couples and is the main redox couple utilized in liquid TGCs. It exhibits a relatively high ionic thermopower of ∼1.4 mV K^−1^ along with favorable interfacial kinetics and ion transport, resulting in superior thermopower density.^67,68^ Research efforts during the past decade in the field of TGCs predominantly concentrated on optimizing the usage of this redox couple and the exploration of alternative electrode materials. For example, by replacing expensive Pt electrodes, which are often utilized to facilitate the interfacial kinetics of the ferri/ferrocyanide couple, with an array of cobaltous oxide nanowires on carbon cloth fiber.^69^ Additionally, alternative redox couples have been explored in recent years to provide greener alternatives, for example the H^+^/H_2_ couple or the Fe^2+^/Fe^3+^ couple.^63,70^ As TGCs continue to evolve and mature, they hold great promise in helping address the growing demand for sustainable energy solutions, particularly in scenarios where waste heat is abundant. The ongoing research and development in this field aims to further optimize system efficiency, materials, and integration techniques to make thermogalvanic systems a significant player in the future of renewable energy conversion technologies. Based on the state of the electrolyte matrix, TGCs can be distinguished into 3 types: liquid or solution based TGCs, solid TGCs and quasi-solid TGCs.

Solution based TGCs represent a category of thermogalvanic systems where the electrolyte matrix is in a liquid state, typically water-based and predominantly centered around the (Fe(CN)_6_^3−^/Fe(CN)_6_^4−^) redox couple.^71^ These cells leverage the dynamic properties of aqueous electrolytes to facilitate ion transport and electrochemical reactions, making them a versatile and practical choice for various applications. They present distinct advantages, such as high ionic conductivity, cost-effectiveness, and utilization of common elements. Their main disadvantage is their limited operational temperature range.^64^ Recently, research on this type of TGCs has focused on improving efficiency by introducing alternative electrode materials or altering the electrolyte matrix.^41,69^ This type of cell is ideal for scenarios where the use of other types of TGCs would be obstructed by cost or constraints related to their materials, such as for harnessing low-temperature heat sources. Aqueous state thermogalvanic cells, leveraging the advantages of water-based electrolytes, present a cheap and versatile solution for various energy conversion applications. Their high ionic conductivity, cost-effectiveness, and ease of handling make them well-suited for both industrial and smaller-scale scenarios with low-temperature heat sources.^72^ Solid-state thermogalvanic cells represent another subset of thermogalvanic systems that differ from their liquid-state counterparts by employing solid-state materials as their electrolyte matrix, these materials can be conductive ceramics, metals, polymers, or other solid compounds with high ionic conductivity, allowing for the transport of ions within the cell. Historically, solid-state thermogalvanic cells based on beta-alumina solid electrolytes garnered significant attention, particularly in aerospace applications.^73^ Despite their inherent advantages, such as heightened stability, enhanced design flexibility, and reduced maintenance demands, these cells have been hindered by their requirement for elevated operating temperatures, rendering them impractical for low-grade heat harvesting applications.^74^ However, recent research endeavors have aimed to address this limitation by exploring the development of solid-state thermogalvanic cells suitable for low-grade heat harvesting purposes.^70^ Similar to their liquid counterparts, solid-state thermogalvanic cells rely on the establishment of thermal gradients across the cell. When one side of the cell is exposed to a higher temperature than the other, the solid electrolyte allows charge transport to occur through ion migration, leading to an electric potential difference between the electrodes.^74^ Solid-state thermogalvanic cells present a viable option, particularly in environments where the use of liquid electrolytes is unfeasible, such as high-temperature conditions. Despite inherent limitations, they remain a significant avenue in the field of thermogalvanic systems, offering robust, efficient, and versatile solutions for diverse energy conversion applications. Quasi-solid thermogalvanic cells are a distinct and relatively new class of thermogalvanic systems that combines the characteristics of solid-state and liquid-state thermogalvanic cells. These cells use a hydrogel or gel-like electrolyte, often in combination with liquid components or ions, to enable efficient ion transport and electrochemical reactions, and have seen the most innovation and research in recent times in the field of TGCs.^75,76^ This hybrid design brings together the benefits of both solid and liquid systems, providing advantages in terms of performance, design flexibility, stability, and ease of fabrication. In these types of systems, the solid-state component of the electrolyte often acts as a scaffold for ion transport, while the liquid or gel-like component carries mobile ions and facilitates electrochemical reactions.^77^ The combination of solid and liquid/gel components in the electrolyte allows for efficient ion transport, which can improve the overall performance of the cell.^78^ Additionally, they offer a degree of flexibility in design, allowing for customization to match the specific requirements of different applications.^39^ Their quasi-solid state provides enhanced stability and resistance to thermal and mechanical stresses, while maintaining high ionic conductivity. This combination leads to greater overall stability in various operating conditions and high thermoelectric performance.^79^ The following Table 1 shows recently reported TGCs, showcasing that an abundant majority of reported TGCs in recent times are either liquid or quasi-solid.

**Table 1:** Summary of recent studies on thermogalvanic cells

Electrolyte state	Matrix	Redox couple	Ionic thermopower (mV K^−1^)	Carnot-relative efficiency	*P*_max_ (W m^−2^)	Reference
Liquid	Water/CsCl	Fe(CN)_6_^3−^/Fe(CN)_6_^4−^	−1.50	—	0.15	72
Solid	Nafion®117	H^+^/H_2_	0.53	0.04%	0.2	70
Quasi-solid	Sand/water	Cu/Cu^2+^	8.40	4%	0.06	75
Quasi-solid	Gelatin/KCl	Fe(CN)_6_^3−^/Fe(CN)_6_^4−^	17.00	—	0.53	39
Liquid	Water/CoO	Fe(CN)_6_^3−^/Fe(CN)_6_^4−^	—	14.80%	24.50	69
Quasi-solid	PVA/GdmCl	Fe(CN)_6_^3−^/Fe(CN)_6_^4−^	6.50	2.66%	1.72	79
Liquid	Water/GdmCl/urea	Fe(CN)_6_^3−^/Fe(CN)_6_^4−^	4.20	0.79%	—	41
Quasi-solid	PAM/CMC	Fe(CN)_6_^3−^/Fe(CN)_6_^4−^	1.26	—	0.02	77
Quasi-solid	MC/KCl	I^−^/I_3_^−^	9.62	—	—	78
Quasi-solid	PVA/PEDOT:PSS	SO_4_^2−^/SO_3_^2−^	1.63	—	—	76

## 5. Thermally regenerative electrochemical cycle

Ionic thermoelectric cycling mode or thermally regenerative electrochemical cycle (TREC) and thermogalvanic technologies are both employed for harvesting low-grade heat, but they operate on distinct principles. Thermogalvanic systems generate electrical energy directly from a temperature gradient across an electrolyte, utilizing the Seebeck effect to create a voltage difference between two electrodes immersed in the electrolyte. However, these systems face significant limitations due to the low ion conductivity of existing electrolytes, which restricts their efficiency to less than 0.5%.^80,81^ In contrast, TREC utilizes a cyclical process to convert thermal energy into electrical energy by exploiting the temperature-dependent voltage of electrodes in a thermodynamic cycle. The TREC approach also addresses some of the limitations of thermogalvanic methods by incorporating mechanisms to manage internal resistance and heat recuperation, making it a more versatile and efficient solution for low-grade heat harvesting.^82^

### 5.1 Working mechanism

**Fig. 10:**
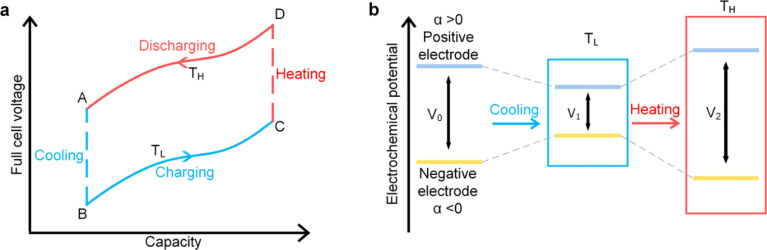
(a) Working mechanism of TREC system for thermal energy harvesting, (b) electrochemical potential change of the electrodes. Reproduced with permission.^81^ Copyright 2019, ACS Publications.

This cycle involves four distinct steps: cooling, charging, heating, and discharging, where the voltage of the cell changes with temperature fluctuations (Fig. 10). In a TREC system, the process begins with a battery-like cell being heated from a low temperature (*T*_L_) to a high temperature (*T*_H_) in an open-circuit state, during which its open-circuit voltage (OCV) varies with temperature changes. The cell is then charged at *T*_H_, absorbing heat and increasing its entropy through electrochemical reactions. After charging, the cell cools back to *T*_L_, causing the OCV to rise. During discharge at *T*_L_, the cell releases heat into the environment, which lowers the voltage. The efficiency is determined by the voltage difference between charging and discharging phases, with key factors including the electrodes' temperature coefficients, their charge capacities, and the system heat capacity.

One of the first research on TREC systems has revealed promising advancements in low-grade heat energy harvesting. Utilizing solid CuHCF and Cu electrodes, the study achieved a cycle efficiency of 3.7% between 10 and 60 °C without heat recuperation and up to 5.7% with 50% heat recuperation.^83^ These efficiencies are notable improvements over previous thermogalvanic cells, which typically had efficiencies below 0.5% at similar temperatures. Another study by Yang *et al.* have developed innovative TREC that efficiently converts low-grade heat into electricity without external electrical charging, enhancing practicality and cost-effectiveness.^80^ Utilizing a Fe(CN)_6_^3−^/_4_^−^ redox pair and a Prussian blue electrode, the system operates between 20 and 60 °C, achieving up to 2% heat-to-electricity conversion efficiency. This cycle discharges at both high and low temperatures with polarity reversal, making it ideal for remote and off-grid settings. Further research is needed to optimize efficiency, stability, and material compatibility. This technology holds promise for extracting low-grade heat from the environment, potentially opening doors for various applications.

In another study, a novel TREC for low-grade heat harvesting was developed using spinel lithium manganese oxide (LMO) and copper hexacyanoferrate (CuHCF) electrodes.^81^ This system operates in a hybrid aqueous electrolyte of lithium and potassium ions, offering a cost-effective and simplified approach compared to traditional methods that require complex ion-exchange membranes. LMO, known for its high positive thermopower of approximately 0.48 mV K^−1^, was successfully paired with CuHCF, which has a negative thermopower of about −0.44 mV K^−1^. The combination of these materials results in a full cell with an overall thermopower of 1.16 mV K^−1^. This system demonstrates a heat-to-electricity conversion efficiency of 1.8% when heat recuperation is not considered, and up to 2.6% with 50% heat recuperation efficiency. The stable electrochemical performance of LMO and CuHCF in the hybrid electrolyte and the high coulombic efficiency observed suggest significant potential for positive-thermopower materials in enhancing low-grade heat harvesting.

In a latest recent research, TREC systems incorporating a nickel hexacyanoferrate (NiHCF) cathode and a zinc anode achieved a notable thermopower of −1.575 mV K^−1^, along with a heat-to-electricity efficiency of 2.41% at a 30 °C temperature difference, equivalent to 25.15% of Carnot efficiency.^82^ This performance surpasses that of existing TREC systems. A key innovation in this work was the introduction of mixed membranes with mixed pH electrolytes, which boosted thermopower to a record-high value of −2.270 mV K^−1^. These advancements in TREC not only showcase significant improvements in converting low-grade heat to electricity but also highlight the potential for further refinement and application in diverse settings, potentially transforming energy harvesting and sustainability efforts.

## 6. Combined ionic thermoelectric devices

Both ionic thermoelectric systems (thermodiffusion-based and thermogalvanic-based) present promising characteristics, yet they have distinct limitations. Thermodiffusion-based systems have excellent mechanical properties and exhibit impressive thermopower, often in the range of several mV K^−1^. However, these systems rely on ion transport and exhibit characteristics analogous to capacitance behaviour. This results in a non-continuous operation mode that can be a disadvantage in applications requiring a constant and consistent energy supply, leading to potential interruptions in power generation or temperature regulation.^84^ Additionally, these devices experience rapid instantaneous power decay during the discharging stage, making long-term continuous operation challenging. This limitation leads to relatively low average power density and energy density, thereby severely limiting their practical applications.^43^

On the other hand, thermogalvanic systems display significant ionic thermopower, surpassing those of traditional electronic thermoelectric materials. Additionally, thermogalvanic cells generate continuous electricity by utilizing temperature gradients, relying on spontaneous electrochemical redox reactions at electrode interfaces. However, conventional thermogalvanic cells due to the liquid nature of the electrolytes, pose encapsulation issues, often leading to leakage problems.^85^ Such vulnerabilities hinder their scalability and reliability, limiting their widespread adoption in practical devices. Developing materials that possess both high thermopowers for efficient energy conversion and mechanical resilience to withstand diverse environmental conditions is a significant hurdle. Additionally, ensuring long-term stability and compatibility with various operating environments is another pressing concern. The quest to overcome these limitations has driven researchers toward exploring the combined potential of thermodiffusion and thermogalvanic effects.^48^ By employing the combined strengths of these mechanisms, researchers aim to develop materials that not only exhibit superior thermopower but also boast enhanced mechanical properties, paving the way for versatile, durable, and efficient ionic thermoelectric materials.

The first such study that investigated the combined potential of both ionic thermoelectric mechanisms was published by Han *et al.* in 2020.^31^ The ionic thermoelectric material, derived from a gelatin matrix modified with ion providers (KCl, NaCl, and KNO_3_) for thermodiffusion and [Fe(CN)_6_^4−^/Fe(CN)_6_^3−^] redox couple for thermogalvanic effect, exhibited an outstanding ionic thermopower of 17 mV K^−1^. This achievement was attributed to the combined interplay between gelatin-KCl (6.7 mV K^−1^) and gelatin–FeCN^4−^/^3−^ (4.8 mV K^−1^). The ionic concentration differences between K^+^ and Cl^−^ (thermodiffusive ions) and K^+^ and FeCN^4−^/^3−^ (redox couple) were also increased due to the negatively charged surface of the gelatin matrix. Consequently, the concentration difference between cations and anions accounted for 71.9% of the total thermopower. The maximum output power of 0.66 mW m^−2^ K^−2^ and harvested energy density of 12.8 J m^−2^ were obtained in the ionic thermoelectric gel gelatin–KCl–FeCN^4−^/^3−^. When wearing a device comprising 25 units (each measuring 5 × 5 × 1.8 mm) connected in series to harness human body heat, a voltage of 2.2 V and a maximum output power of 5 μW were attained. This foundational study highlighted the promising approach of exploiting the combined effects of thermogalvanic and thermodiffusion mechanisms to significantly enhance the ionic thermopower of quasi-solid electrolytes, paving the way for efficient and leakproof ionic thermoelectric devices.

### 6.1 Optimisation parameters

Over the last three years, there has been significant progress in the study of combined ionic thermoelectric materials. Researchers have been working to improve three crucial aspects: ionic thermopower (mV K^−1^), which relates to the material's ability to convert heat into electricity; output power density (W m^−2^), indicating how much power can be generated per unit area; and mechanical properties, ensuring the durability and stability of devices.^86^ To achieve these goals, scientists have explored various combinations of polymer matrices, thermodiffusive electrolytes, redox pairs, and electrode materials. The main aim of these studies is to find the most effective combination that enhances the ionic thermoelectric efficiency and durability of ionic thermoelectric materials. The findings from these studies, detailed in Table 2, provide valuable insights into the ongoing efforts to harness the potential of these materials.

**Table 2:** Summary of recent studies on combined ionic thermoelectric devices

Polymer	Electrolyte	Redox pair	Seebeck coefficient (mV K^−1^)	Ionic/electrical conductivity (mS cm^−1^)	Thermal conductivity (W m^−1^ K^−1^)	Specific power density (mW m^−2^ K^−2^)	Energy density (J m^−2^)	Ref.
Gelatin/glutaraldehyde	KCl	FeCN^4−^/^3−^	24.7	—	—	9.6	198	87
Gelatin	KCl	FeCN^4−^/^3−^	17	—	—	8.9	80	39
Acrylic acid-acrylamide/carboxymethyl cellulose	H_2_SO_4_	Polyaniline	40.60	30.2	0.4551	11.31	570	38
Polyacrylamide/carboxymethyl cellulose	Li_2_SO_4_	FeCN^4−^/^3−^	11.58	18.4	0.47	32 mW m^−2^	—	2
Polyvinyl alcohol/gelatin	NaCl	Fe^2+^/Fe^3+^	−1.63	7	0.6	1.2 mW m^−2^	—	88
Gelatin	KCl	FeCN^4−^/^3−^	17	5 mS m^−1^	0.15	0.66	12.8	31
Bacterial cellulose	LiBr	FeCN^4−^/^3−^	1.21	—	—	≈0.03	—	85
Polyvinyl alcohol	GdmCl	FeCN^4−^/^3−^	6.5	≈15	0.473	1.96	—	79
Polyvinyl alcohol	NaCl	Sn^2+^/Sn^4+^	−1.62	≈9	2.08	2.8 mW m^−2^	160 mJ m^−2^	89
Polyvinyl alcohol	NaCl	SO_4_/_3_^2−^	1.63	29.2	0.7	72 nW	—	76
Methylcellulose	KCl	I^−^/I_3_^−^	9.62/−8.18	6	—	0.36/0.12	—	78
Polyacrylamide/sodium alginate	GdmCl	FeCN^4−^/^3−^	4.4	105	≈0.55	1.78	—	90

### 6.2 Selection of polymer matrices

**Fig. 11:**
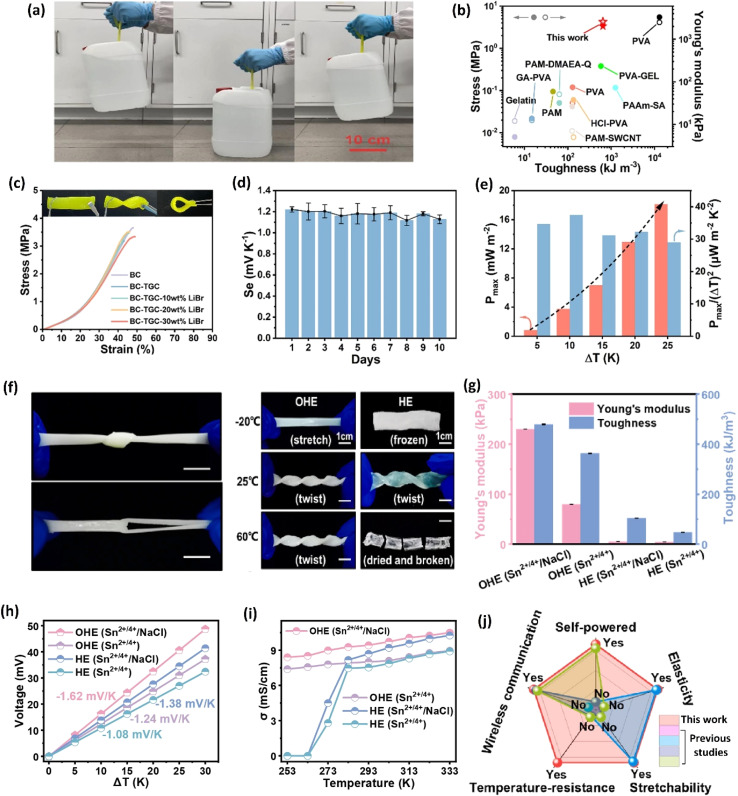
Bacterial cellulose–LiBr–FeCN^4−^/^3−^ hydrogel: mechanical properties (a) hydrogel holding 5 kg water bottle, (b) toughness and Young's modulus, (c) stress–strain curves; ionic thermoelectric properties (d) ionic thermopower variations over the period of 10 days, (e) output power density *vs.* temperature difference, reproduced with permission.^85^ Copyright 2023, Elsevier; organohydrogel electrolyte: (f) mechanical properties of hydrogel in tensile deformation including knotting and crossing stretching, (g) photos of the OHE after 6 h at different temperatures, (h) thermovoltage response *vs.* temperature difference, (i) conductivity as a function of temperature, and (j) comparison of this study with previous studies, reproduced with permission.^89^ Copyright 2023, Elsevier.

The selection of a polymer matrix is one of the key parameters for enhancing the performance of ionic thermoelectric devices based on their surface functionalities. Polymers with suitable surface properties enhance ionic diffusion, a key factor in improving the material's thermoelectric performance.^16^ Additionally, selected polymers should exhibit excellent mechanical properties, ensuring the resulting ionic thermoelectric device stability and durability. The selection criteria for such polymers are rooted in their chemical composition. For instance, polyvinyl alcohol (PVA) is valued for its excellent ability to create a stable matrix and superior mechanical properties.^91^ Its chemical structure, featuring hydroxyl groups, provides numerous functional sites, facilitates strong hydrogen bonding, which enhances the mechanical integrity and flexibility of the resulting materials. This is crucial for ionic thermoelectric devices as it allows the matrix to accommodate ionic movement without structural degradation. Moreover, its hydrophilic nature supports the incorporation and uniform distribution of ionic species, enhancing ionic conductivity and thermoelectric performance. Li and team successfully prepared an organohydrogel electrolyte (OHE) using a straightforward freezing–thawing approach, incorporating PVA polymer, Sn^2+^/Sn^4+^ redox couple, and NaCl as a thermodiffusive ions electrolyte.^89^ This OHE exhibited exceptional flexibility and mechanical strength, attributed to the properties of the PVA polymer, enabling it to withstand diverse deformations (Fig. 11a–c). Remarkably, the OHE displayed anti-freezing and anti-drying characteristics, making it operatable effectively across a broad temperature range (−20 to 60 °C). Moreover, the OHE demonstrated outstanding ionic thermoelectric properties, including a remarkable ionic thermopower of −1.62 mV K^−1^, a high figure of merit (3.75), and an impressive maximum power density of 2.8 mW m^−2^ (Fig. 11d and e). These findings highlight the potential of PVA-based OHE's for practical applications, emphasizing its robust performance and adaptability in demanding environments.

Gelatin, derived from collagen, possesses unique biopolymer characteristics, making it biocompatible and environmentally friendly.^92^ It possesses a triple-helix structure that provides good mechanical strength and flexibility. The ability of gelatin to form hydrogels with high water content improves ionic mobility, which is essential for efficient ionic thermoelectric conduction. The amphoteric nature of gelatin, containing both carboxyl and amino groups, allows for effective ionic exchange and interaction with various ionic species, thus enhancing the thermoelectric efficiency of the device.^93^ A quasi-solid-state ionic thermoelectric cell, fabricated by incorporating glutaraldehyde into a gelatin–KCl–FeCN^4−^/^3−^ matrix, exhibited significantly enhanced thermoelectric properties.^87^ The resulting robust, porous structure increased the maximum temperature difference from 9 °C to 23 °C and boosted thermopower (24.7 mV K^−1^) due to expanded entropy between redox couples. This led to a record-high power density of 9.6 mW m^−2^ K^−2^ and a 2 hours energy density of 198 J m^−2^. Demonstrating excellent cycling stability and generating a high voltage of 3.6 V and 115 μW output in a wearable device, this cell showcases promising potential for practical applications.

Cellulose, composed of glucose units, offers high mechanical strength and chemical stability.^94^ The extensive hydrogen-bonding network within cellulose provides significant tensile strength and rigidity, which helps in maintaining the structural integrity of ionic thermoelectric devices under mechanical stress. Additionally, the hydrophilic nature of cellulose facilitates the absorption and transport of ionic species, which is critical for maintaining high ionic conductivity. The abundance of hydroxyl groups in cellulose also allows for functionalization, which can be tailored to improve specific ionic interactions and enhance thermoelectric properties.^95^ In a recent study conducted by Yin *et al.* in 2023, it was demonstrated that bacterial cellulose also has the potential to be utilized in creating quasi-solid ionic thermogalvanic cells.^85^ These cells exhibited impressive mechanical characteristics and thermoelectric properties, such as toughness, elasticity, flexibility, ionic conductivity, ionic thermopower, comparable to those found in PVA-based devices (Fig. 11f–j). They employed FeCN^4−^/^3−^ redox couple along with LiBr hygroscopic salt to give superior anti-freezing and anti-drying properties to prepare thermogalvanic cells. The prepared cells exhibit an excellent ionic thermopower of 1.62 mV K^−1^, which remains at 1.06 mV K^−1^ after three months of air exposure, indicating their high stability (Fig. 11h).

Polyacrylamide (PAAM) is recognized for its ability to form hydrogels with high water content, significantly enhancing ionic mobility and overall ionic conductivity. The hydrophilic nature of PAAM allows it to absorb large amounts of water, creating a conducive environment for the movement of ions, which is critical for improving the thermoelectric performance of devices. In a notable study, Chen *et al.* successfully developed a water-resistant ionic thermoelectric gel by integrating the hydrophobic structure of PMMA with a hydrophobic ionic liquid (BMIM:PF_6_).^96^ This innovative combination resulted in an i-TE gel that achieved an impressive thermopower of 3.1 mV K^−1^. The incorporation of PMMA provided the necessary mechanical strength and stability, while the hydrophobic ionic liquid ensured efficient ionic transport and enhanced thermoelectric properties.

Agarose, a seaweed-derived linear polysaccharide, forms thermally stable gels, ideal for applications demanding consistent performance across temperature fluctuations. Its hydrophilic nature readily accommodates water molecules within its structure. Simple agarose based hydrogels proves not suitable for ionic thermoelectric devices.^97^ Recent research, however, has demonstrated the potential of agarose-based i-TE gels. By incorporating sodium dodecyl benzene sulfonate (DBS), a remarkable p-type thermopower of 41.8 mV K^−1^ was achieved.^98^ This significant enhancement stems from the formation of a unique porous structure through DBS micellization. The hydrophilic sulfonic group binds to the agarose gel, while the hydrophobic alkyl chain orients inward, immobilizing DBS-ions relative to sodium ions. This structural decoupling of thermodiffusion markedly improves i-TE performance.

Polyurethane (PU) can also be a useful polymer for ionic thermoelectric applications due to its excellent mechanical properties and structural diversity. A latest study developed a PU ionogel with self-healing, high thermoelectric performance, transparency, and stretchability, making it ideal for wearable thermoelectric generators.^99^ The ionogels, synthesized using PU prepolymer, a reversible borate bond crosslinker, and an ionic liquid (EMIM:DCA), showed high thermovoltage (up to 25.6 mV K^−1^) and significant stretchability (up to 635% strain at break). Self-healing restored 95% of tensile strength and 99% strain at break within 30 minutes. The ITESC fabricated a maximum energy output of 2.34 μW m^−2^ with an 8 kΩ load at a 0.4 K temperature difference, highlighting their potential for durable, efficient, and flexible wearable energy solutions.

Poly(vinylidene fluoride) (PVDF) is another non-aqueous polymer known for its high chemical resistance, thermal stability, and mechanical strength, making it suitable for ionic thermoelectric applications. A recent study demonstrates a significant improvement in the thermopower of PVDF-HFP ionogels by doping with sodium dicyanamide (Na:DCA).^100^ The ionogel doped with 0.5% in mol of Na^+^ relative to EMIM^+^ achieved a thermopower of 43.8 mV K^−1^, ionic conductivity of 19.4 mS cm^−1^, and thermal conductivity of 0.183 W m^−1^ K^−1^. These enhanced ionogels are promising for use in iTE capacitors for thermoelectric conversion.

Sodium alginate, extracted from brown algae, boasts excellent ion exchange capabilities due to its polysaccharide nature.^101^ The presence of carboxyl groups in its molecular structure enables strong ionic interactions and exchange, which are vital for ionic thermoelectric performance. This characteristic, combined with its good biocompatibility and mechanical flexibility, makes sodium alginate an ideal candidate for enhancing the ionic conductivity and overall thermoelectric efficiency of devices. These inherent chemical attributes make these polymers highly suitable for synthesizing quasi-solid ionic thermoelectric devices, meeting the criteria of both enhanced ionic diffusion and mechanical stability, thus ensuring the device's robustness and effectiveness in real-world applications.

These promising advances suggest that the future of this field will involve exploration of the possibility of combining two or more polymers in a single matrix. By integrating the unique properties of each polymer, scientists might create innovative quasi-solid matrices with enhanced flexibility, durability, superior mechanical strength, and remarkable ionic thermoelectric properties. This novel approach may open new horizons in the development of combined ionic thermoelectric devices for efficient energy harvesting applications.

### 6.3 Selection of thermodiffusive electrolytes

Another vital aspect for optimising the performance of combined ionic thermoelectric devices involves the selection of suitable inorganic/organic salts as thermodiffusive electrolytes. Salts like NaCl, KCl, LiBr, GdmCl, Li_2_SO_4_, and H_2_SO_4_ have been employed in recent years owing to their easy dissolution in solvents and high conductivity. Easy dissolution is crucial for the uniform mixing of salts within the polymer matrix, ensuring a consistent distribution of the electrolyte throughout the quasi-solid gels.^102^ The high conductivity of these salts enables efficient ion transport within the material, a fundamental requirement for enhancing ionic thermoelectric performance.^8^ The careful selection of these inorganic salts is therefore a cornerstone for advanced combined ionic thermoelectric devices to achieve exceptional levels of efficiency and practicality.

**Fig. 12:**
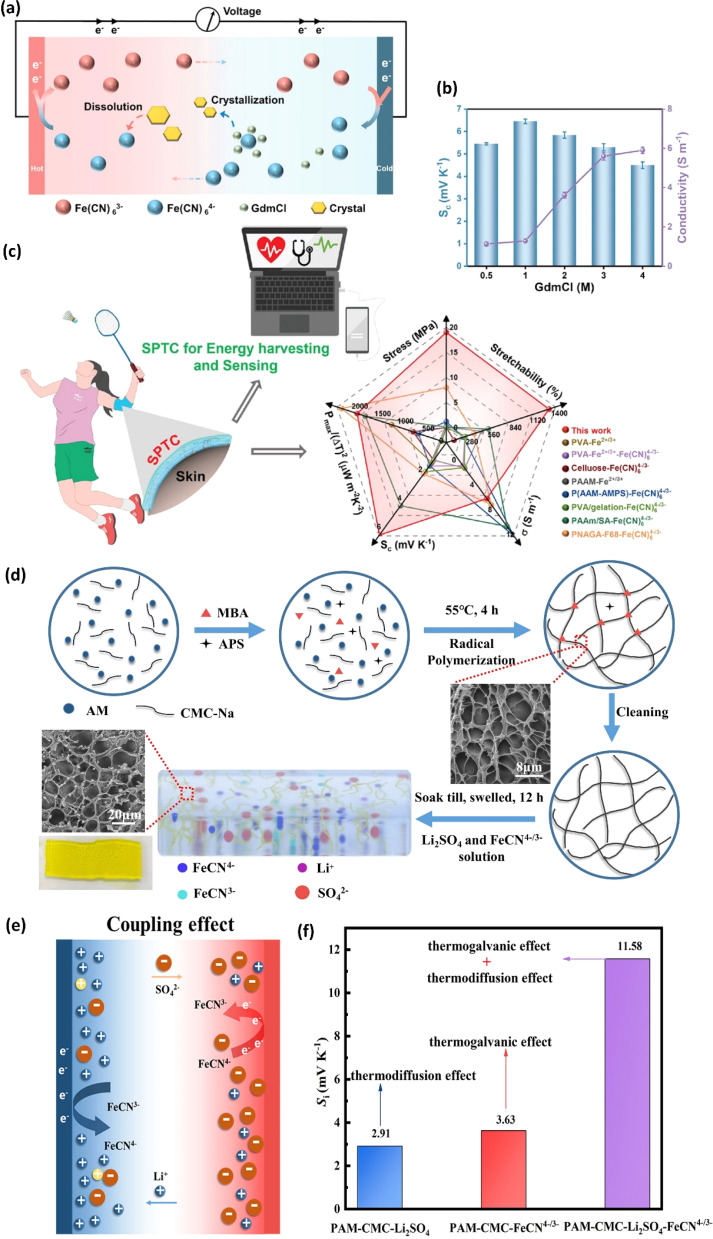
PVA–GdmCl–FeCN^4−^/^3−^ based thermogalvanic hydrogel: (a) schematic illustration of the Gdm^+^ contribution to the thermogalvanic effect, (b) Seebeck and conductivity as function of electrolyte concentration, (c) schematic diagram of employing the device to power up the medical devices and the comprehensive comparison with previous quasi-solid stretchable thermogalvanic thermocells, reproduced with permission.^79^ Copyright 2023, Wiley-VCH; cellulose–Li_2_SO_4_–FeCN^4−^/^3−^ based thermogalvanic hydrogel: schematic representation of (d) preparation process of hydrogel and its microstructure, (e) mechanism of coupling effect, and (f) ionic Seebeck before and after coupling, reproduced with permission.^2^ Copyright 2023, Elsevier.

Liu *et al.* 2023 fabricated a quasi-solid stretchable thermogalvanic thermocell using PVA as a polymer matrix, FeCN^4−^/^3−^ as redox couple, and guanidinium chloride (GdmCl) as electrolyte.^79^ The use of PVA polymer conveyed excellent mechanical properties to the cells in terms of stretchability (1300%) and toughness (163.4 MJ m^−3^), as shown in Fig. 12c. The inclusion of GdmCl increased the entropy difference in the FeCN^4−^/^3−^ redox couple due to Gdm^+^-induced crystallization of FeCN^4−^ ions, resulting in superior thermogalvanic performance (Fig. 12a). The resulting system had a large thermopower of 6.5 mV K^−1^ and an excellent high specific output power density of 1969 μW m^−2^ K^−2^ (Fig. 12b). Another study published by Zhou *et al.* 2023 prepared a carboxymethyl cellulose-based quasi-solid gel composed of FeCN^4−^/^3−^ redox couple and Li_2_SO_4_ salt as thermodiffusive electrolyte.^2^ Li^+^ ions have a smaller radius and exhibit a faster migration rate in the electrolyte resulting in better ionic diffusion, contributing to the overall performance of the device (Fig. 12d and e). By combining the principles of thermodiffusion and the thermogalvanic effect, the prepared devices demonstrated a superior ionic thermopower of 11.58 mV K^−1^ (Fig. 12f). Moreover, these devices exhibited a high ionic conductivity of 18.4 mS cm^−1^, indicating the rapid migration of Li^+^ ions through the electrolyte, leading to enhanced ionic diffusion. This unique combination resulted in a noteworthy thermoelectric figure of merit of 0.11 and a substantial power factor of 198.2 μW m^−1^ K^−2^ at room temperature, underscoring their exceptional overall performance.

Future research directions in this domain are likely to focus on refining electrolyte selection to further enhance their efficiency and contribute towards their commercialisation. Investigating novel inorganic salts and their combinations could provide insights into improving ionic diffusion and thermoelectric performance. Moreover, the exploration of organic–inorganic hybrid electrolytes might offer a new avenue, potentially combining the advantages of both materials. Additionally, optimising the interplay between electrolyte composition, polymer matrix, and redox couples could lead to the development of advanced quasi-solid gels with superior homogeneity and stability, contributing to prolonged device lifespan. Ultimately, future research activities should explore innovative electrolytes, pushing the boundaries of combined ionic thermoelectric devices and opening avenues for sustainable energy harvesting technologies.

### 6.4 Selection of redox pairs

In the past few years, the exploration of a variety of redox couples and their interplay with the rest of the components has also been a focal point of researchers. Redox reactions, involving the transfer of electrons between matrix and electrodes, are associated with an increase in the entropy of the system that can result in efficient electron transfer.^103^ Therefore, researchers have examined diverse redox couples (FeCN^4−^/^3−^, Fe^2+^/Fe^3+^, Sn^2+^/Sn^4+^, SO_4_/_3_^2−^, I^−^/I^3−^) to influence this increase in entropy. By understanding and manipulating this entropy change, scientists aim to identify optimal redox pairs that can efficiently facilitate electron transfer, thereby enhancing ionic thermoelectric efficiency.

**Fig. 13:**
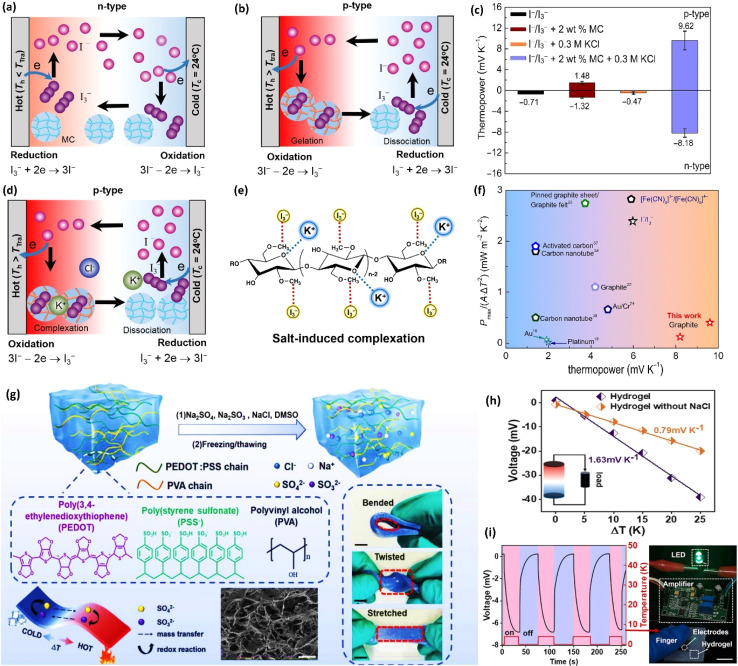
Methylcellulose–KCl–I^−^/I_3_^−^ based thermogalvanic gel: schematics of the polarization switching from (a) n-type to (b) p-type, (c) comparison of the thermopowers, schematics of (d) a p-type TGC with the ternary electrolyte and (e) salt-induced complexation. K^+^ and I_3_^−^ ions, (f) comparison of this study with previous studies, reproduced with permission.^78^ Copyright 2022, *Science*; PVA-NaCl-SO_4_/_3_^2−^ based hydrogel (g) schematic illustration of design, chemical structure, morphology, and mechanical properties, (h) thermal voltage response at different temperature differences, and (i) output voltage of the hydrogel under 4-repeated heating–cooling cycles for powering LED, reproduced with permission.^76^ Copyright 2023, Elsevier.

In a study by Han and colleagues in 2022, thermogalvanic cells were created using a substance called methylcellulose, a redox pair I^−^/I_3_^−^, and KCl as the electrolyte.^78^ Methylcellulose, which responds to temperature changes, resulted in a polarization switching from n-type to p-type above a transition temperature as shown in Fig. 13a and b. This change occurred due to a strong hydrophobic interaction between I_3_^−^ ions and methylcellulose. As a result, the cells exhibited remarkably high thermoelectric voltages of 9.62 mV K^−1^ for p-type and −8.18 mV K^−1^ for n-type (Fig. 13c). These high thermopower values were attributed to the gelation effect of I_3_^−^ ions with methylcellulose at a hot electrode resulting in an increased entropy change and differences in ion concentrations in the redox pair (Fig. 13d and e). The optimized p-type thermogalvanic cell achieved a normalized maximum power density of 0.36 mW m^−2^ K^−2^ (Fig. 13f). A recent study by Tian *et al.* 2023 reported a novel non-toxic redox couple (SO_4_/_3_^2−^) in a PVA matrix along with NaCl electrolyte.^76^ The fabricated device had excellent mechanical properties including stretchability and flexibility because of PVA (Fig. 13g). Moreover, the novel redox pair at a concentration of 0.1 M, resulted in an excellent ionic thermopower value of 1.63 mV K^−1^ (Fig. 13h and i). The addition of thermodiffusive electrolytes led to an improved ionic conductivity of 29.2 mS cm^−1^ which overall enhanced the ionic thermoelectric performance of the devices.

Future research directions emphasize the strategic selection of redox couples. Exploring a broader spectrum of redox pairs beyond traditional ones holds the key to unlocking innovative avenues. Researchers should comprehensively investigate the synthesis and evaluation of novel redox couples, considering factors like electrochemical stability, entropy change, and efficiency in facilitating electron transfer. Furthermore, it would be noteworthy to explore organic-based redox couples and other environmentally benign materials, aiming to reduce the ecological footprint of ionic thermoelectric devices.

### 6.5 Selection of electrode materials

**Fig. 14:**
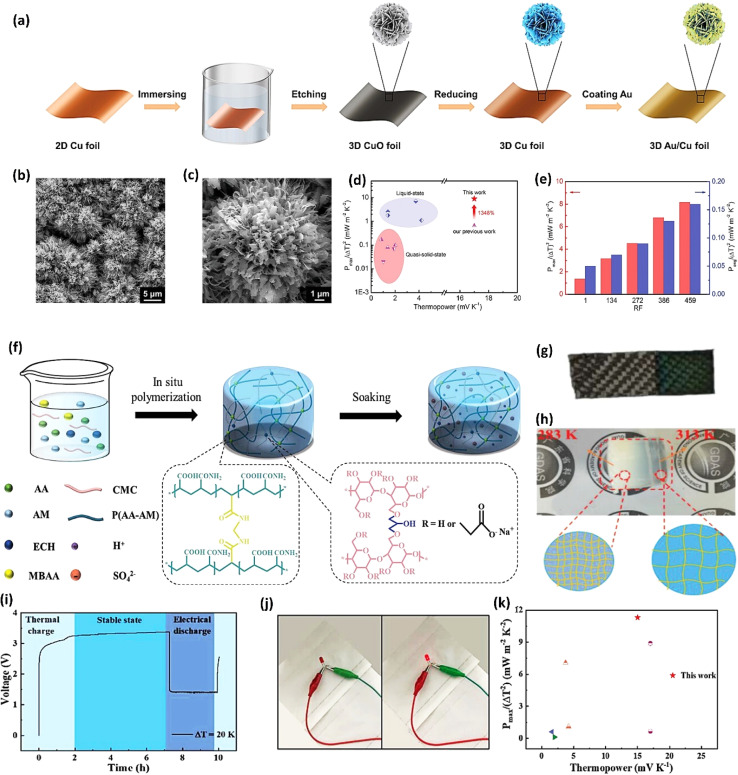
3D Au/Cu electrodes: (a) schematic illustration showing the preparation process of the 3D Au/Cu foil from 2D Cu foil, (b and c) SEM images of as-fabricated microflower CuO foil, (d) thermoelectric performance comparison of this work with previous studies, (e) output power density compared to RF values of electrodes, reproduced with permission.^39^ Copyright 2022, Wiley-VCH; PANI@CWF electrodes with cellulose–H_2_SO_4_ hydrogel: (f) schematic diagram of the preparation process and chemical structure of hydrogel, (g) digital image of PANI@CWF electrodes, (h) schematic diagram of hydrogel networks under Δ*T*, (i) thermal charging and electrical discharging process of the integrated generator, (j) LED light powered by the generator, and (k) comparison of this work with previous studies, reproduced with permission.^38^ Copyright 2023, Wiley-VCH.

A recent advancement in enhancing the power and energy density of combined ionic thermoelectric devices involves the engineering of the device electrodes. In a study conducted by Li and colleagues in 2022, advancements were made in enhancing the power and energy density of combined ionic thermoelectric devices by focusing on electrode engineering.^39^ They employed a quasi-solid ionic thermoelectric device based on gelatin/KCl/FeCN^4−^/^3−^ from their previous study and altered the electrodes.^31^ They developed three-dimensional hierarchical copper electrodes coated with gold (3D Au/Cu) using a specialized oxidization–etching–reduction technique (Fig. 14a). These electrodes exhibited a unique micro flower-like structure, comprising numerous porous and zigzagging petals (Fig. 14b and c). This structure significantly increased the electroactive surface area of the electrodes, providing more sites for thermogalvanic reactions and reducing the resistance at the interface for charge transfer. The modified device demonstrated a comparable ionic thermopower of 17 mV K^−1^ (Fig. 14d). Remarkably, it achieved an exceptional instantaneous output power density of 8.9 mW m^−2^ K^−2^ and an exceptionally high output energy density of 80 J m^−2^ under a temperature difference of 9 K (Fig. 14e). Over a week-long continuous operation, the device maintained an average energy density of 59.4 J per m^2^ per day. These findings represent a significant step forward in the development of efficient and wearable thermoelectric devices.

A recent research study introduced an innovative approach to ionic thermoelectric systems by employing polymer redox polyaniline (PANI) doped carbon weaved fabric electrodes (PANI@CWF) and an ionic gel based on carboxymethyl cellulose (Fig. 14f and g).^38^ This gel featured a distinct interpenetrating polymer network structure, infused with H_2_SO_4_ to enhance hydrogen bonds, thereby improving its mechanical properties and thermopower output (Fig. 14h). This unique system exhibited a remarkable Seebeck of 40.60 mV K^−1^ and achieved a maximum figure of merit value of 3.95 at a temperature difference of 5 K. The study also revealed a synergistic effect of the PANI@CWF electrode, enhancing current density through redox reactions on both electrodes. Consequently, this configuration yielded an output power density of 11.31 mW m^−2^ K^−2^ and an energy density of 570 J m^−2^, demonstrating its significant potential for practical energy harvesting applications (Fig. 14i–k).

## 7. Theoretical models conduction in electrolytes

### 7.1 Organic electronic, ionic and mixed conductors

**Fig. 15:**
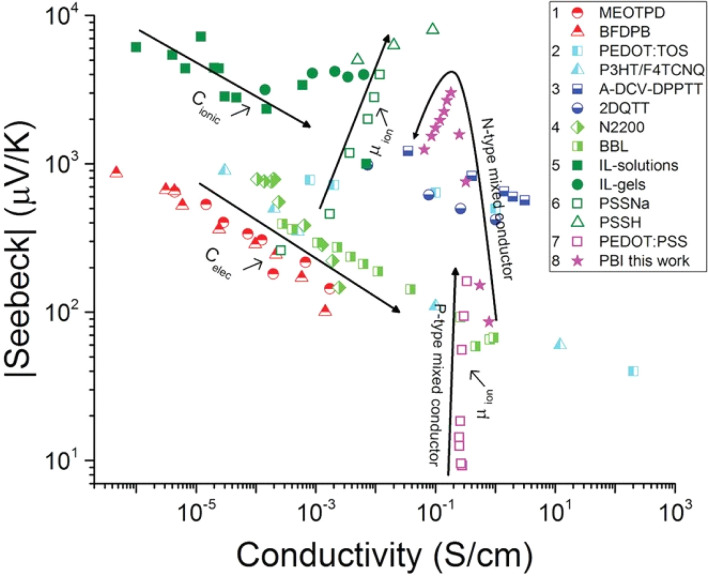
Seebeck coefficient as a function of the electrical conductivity, reproduced with permission.^104^ Copyright 2020, Wiley-VCH.

In Fig. 15 we show a plot of the Seebeck coefficient as a function of the conductivity in a double logarithmic for several electronic, ionic and mixed materials. In most polymers the dependence is *S* ∝ *σ*^1/4^, typical of transport by hopping. However, in PEDOT the dependence is much lower, typical of a semiconductor. The electrical conductivity is *σ* = −*enμ*_*n*_(*epμ*_*p*_), *i.e.* is proportional to the mobility, which in most polymers is of the order of 10^−4^–10^−5^ cm^2^ V^−1^ s^−1^, while in PEDOT is much higher, of the order of 1 cm^2^ V^−1^ s^−1^, thus, the electron (or hole) concentration to have a given conductivity is much lower in PEDOT, giving a semiconducting behaviour. In ionic materials, the ionic thermopower increases with increasing ionic concentration. If in contrast, we have a p-type semiconductor, since the carriers diffuse from the hot region to the colder one, the thermopower will be positive. In the case of electronic conduction, the Seebeck conduction (this is an approximation for metals) can be written as5
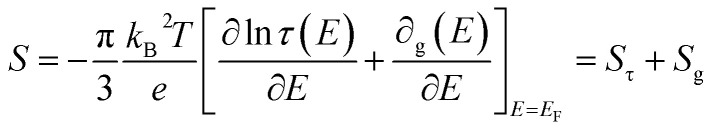


**Fig. 16:**
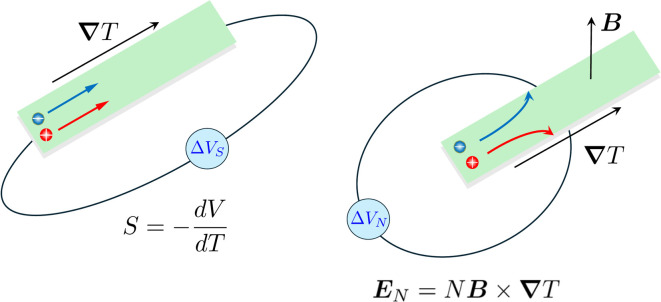
Seebeck and Nernst coefficients, reproduced with permission.^105^ Copyright 2021, American Physical Society.

The Seebeck coefficient has two contributions, one coming from the relaxation time or the mobility, and a second one coming from variations in the density of states. This second term is often suggested as the way to increase the Seebeck coefficient changing the density of states by nanostructuration. The change in the Seebeck coefficient due to the increase of the mobility can be related to the Nernst coefficient, which can be obtained applying a magnetic field perpendicular to the temperature gradient and measuring the voltage in the transversal direction, as shown in Fig. 16. Physically, the Peltier effect is the opposite to the Seebeck effect, if we supply a voltage difference in the two ends of the semiconductor we produce a temperature gradient.

Qiao *et al.*^106^ proposed a linear scheme to solve numerically the Poisson–Nernst–Plank equations. It is based on gradient-flow formulation. They linearize the logarithm function. Fish^107^ examine the nanocomposite electrolyte through a theoretical model. A rigorous description of the space charge layer and its impact on conductivity insulating spheres dispersed in an ionic conducting material.

Fleharty *et al.*^108^ modelled small nanochannel with an electric double layer formed at the walls comparable to the channel width. They solve the Poisson–Boltzmann equation with charge regulation boundary conditions Moya *et al.*^109^ studied cylindrical nanopores filled with a ternary electrolyte solution as a function of the sign of the fixed charge on the pore wall. The Poisson–Boltzmann equation in cylindrical coordinates was solved by using the network simulation method. The velocity and conductivity were obtained using the modified Navier–Stokes equation and the Nernst–Planck flux equations. Koerver *et al.*^110^ used cobalt bipyridyl salts as active redox couple, they used cyclic voltammetry to determine the Seebeck coefficient. Jing *et al.*^111^ analyze the electro-viscous effect on the EDL, it seems that, at sufficiently high *Z* potential, the effect is negligible.

Luo *et al.*^112^ studied the electrophoresis and electric conduction in a salt-free suspension. They obtained analytical expressions for electrophoretic mobility and effective electrical conductivity. The scale *z*-potential, increases phoretic mobility and effective electric conductivity increases monotonically with the increase of the scale surface charge density. In addition, Varner^113^ studied the effect of dilution in ionic liquid supercapacitors. Huang^114^ studed the effect of hydrodynamic slippage on the power generation performance, indicating that velocity boundary conditions are relevant at the nanoscale. Levy *et al.*^115^ studied the variation of the dielectric response of the ionic liquid including the interaction of the ions with the dipoles present in the liquid. A closed form for the dielectric constant is obtained. Stout *et al.*^116^ have written the basic principles of ionic transport, the basic equations, *etc.* Qiao *et al.*^106^ developed a linear scheme for the solution of the Poisson–Nernst–Planck equations.

### 7.2 Basic formulation of the problem

#### 7.2.1 One dimensional case

The first calculations of the thermopower in a thermocell filled with an electrolyte were supplied by Eastman^117^ and Wagner^118^ They found, for the stationary state of the thermopower or Seebeck coefficient, the expression6
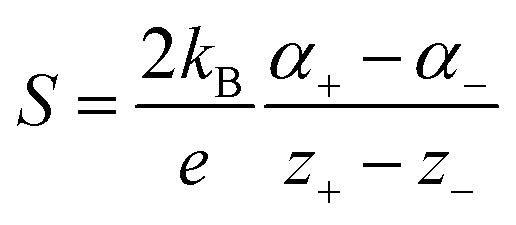
where *k*_B_ is the Boltzmann constant, *e* the elementary charge, *α* the Soret coefficients and *z* the ionic charge.

**Fig. 17:**
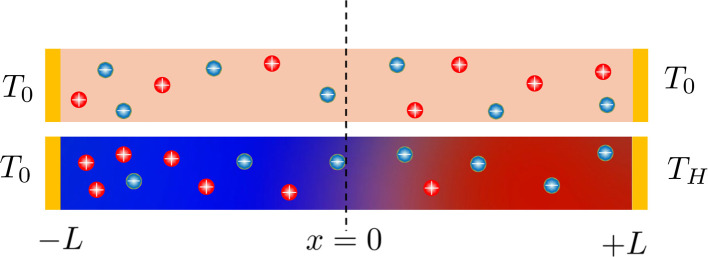
Upper panel: electrolyte with the ions in thermal equilibrium; lower panel: ion distribution when a temperature gradient exists through the electrolyte.

The basic problem is that of a channel ended in two electrodes as drawn in Fig. 17, with an electrolyte with anions and cations. In the upper panel of Fig. 17 we show the channel at equilibrium at a temperature *T*_0_ with a completely dissociated electrolyte solution. Let us call *n*_+_ the concentration of cations and *n*_*−*_ that of anions. The lower panel shows the ion distribution when a temperature gradient (*T*_0_ → *T*_H_, *T*_H_ being the temperature at the hot side) is applied through the electrolyte. We are supposed to have a long channel with a length of 2*L*. When compared with the lateral dimensions *d* (*d* ≪ *L*), but *d* is assumed to be wide enough to consider the problem as one dimensional. The mass and charge conservation are given by the continuity equation, which in the case of charged particles moving in a fluid is called the Nernst–Planck equation,7
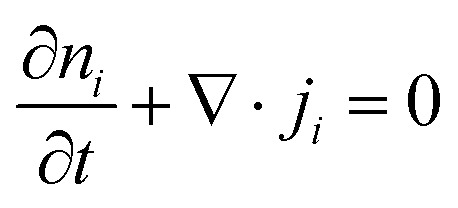
where *n*_*i*_(*r*, *t*) is the concentration of particles *i* and *j*_*i*_(*r*, *t*) the flux of the charged particles. In the simplest case, there will be two Nernst–Planck equations. The general expression for the flux corresponding to particle *i* is8
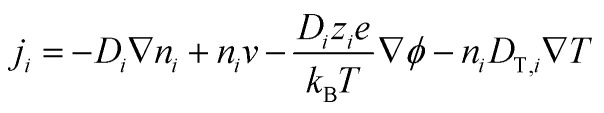
where *D*_*i*_ is the Brownian diffusion coefficient,^119^ solution of the diffusion equation9
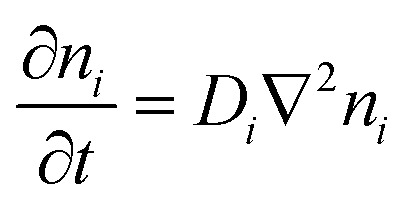


*D*_*i*_ can be written as *D*_*i*_ = *μ*_*i*_*k*_B_*T*, the so called Einstein relation, *μ*_*i*_ being the ion drift mobility, the second term is the contribution of the fluid movement (advection), *v*(*r*, *t*) being the fluid velocity, the third term is the contribution to the ionic current due to the existence of an electric field *E* = −∇*ϕ* (electromigration), the fourth is the thermodiffusive flux, *D*_T,*i*_ being the thermodiffusion coefficient. The Brownian diffusion coefficient is assumed to be the same for anions and cations and the thermodiffusion coefficient can be written in terms of the thermodiffusion factor *α*_*i*_, sometimes called Soret coefficient (actually *α* = *S*_T_*T*, *S*_T_ being the Soret coefficient^51^).10
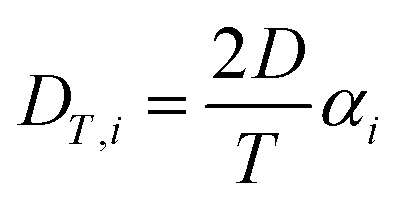


Thus, in the one-dimensional case, assuming that the fluid is at rest, the ionic fluxes become^116,120^11
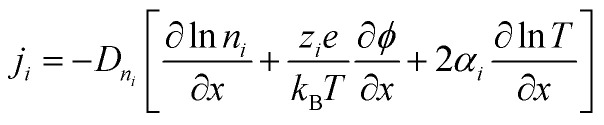


On the other hand, the electrostatic potential must satisfy the Poisson equation,12
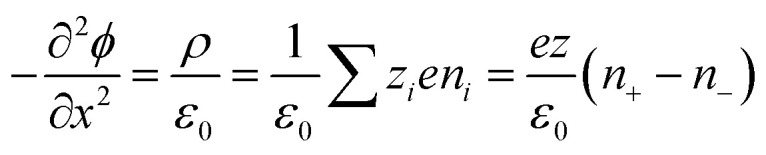
assuming that |*z*_+_| = |*z*_−_|. Finally, the temperature has to be subjected to the heat equation13
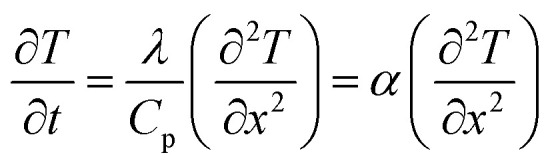
where *C*_p_ is the volumetric heat capacity (J K^−1^ m^3^) at constant pressure and *λ* (W m^−1^ K^−1^) is the thermal conductivity of the electrolyte, *α* (m^2^ s^−1^) is the thermal diffusivity.

The thermovoltage can be calculated from14
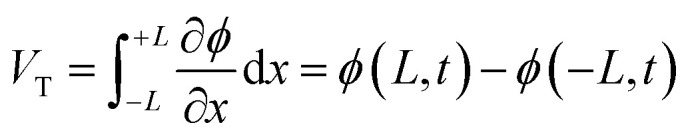
it depends only on the potential difference in the one-dimensional case.

To proceed further some approximations are needed. We will assume that all transients have already ended, and the temperature gradient is small (Δ*T* = *T*_H_ – *T*_0_ ≪ *T*_0_). Then, the ion concentration *n*_*i*_ = *n*_*i*,0_ + *δn*_*i*_, where *n*_*i*,0_ is the equilibrium ion density. If *D*/*α* ≪ 1 (*D*/*α* ≈ 100 for aqueous solutions of electrolytes^116,121^), we can neglect the temporal dependence (basically we can consider that the temperature changes instantaneously from the initial to the final value) and the solution of eqn (13) is15
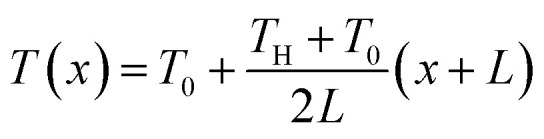


Then, the thermodiffusive contribution to the current has been reduced to a constant16
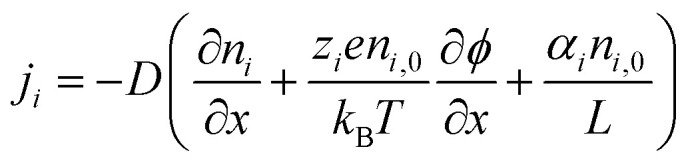


Introducing *j*_*i*_ in the Nernst–Planck equation, Stout R. F. *et al.*^116^ analyze the dynamics of the ionic charges within the electrolyte under low temperature gradients using the Laplace transformation17
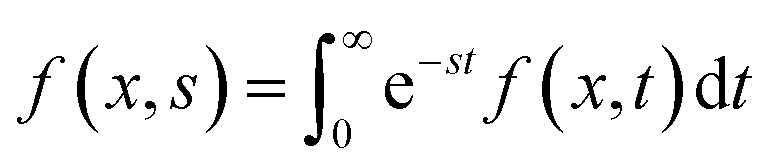
where *s* = *σ* + *iω*. The Laplace transform is used to transform the time domain equations in frequency domain. It is more general than the Fourier transform and can have solutions in equations where the Fourier transform does not. Defining18
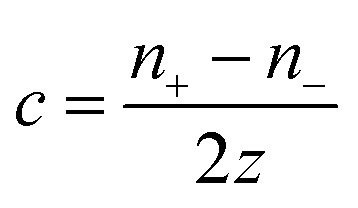
as the neutral salt concentration and19
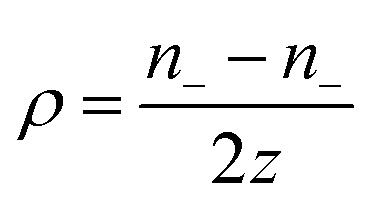


The charge density is divided by the elementary charge. Using the appropriate boundary conditions,^116^ the solution of these equations are
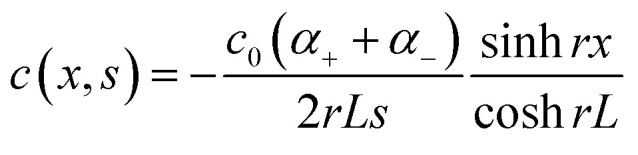

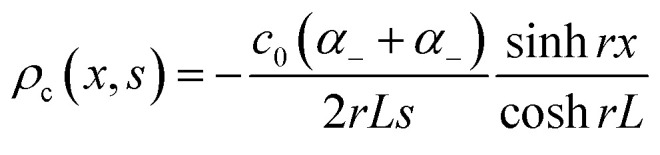
20
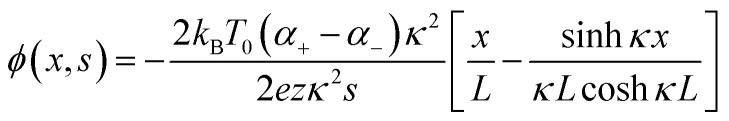
where 1/*κ* = ^p^*ε*_0_*k*_B_*T*_0_/2*e*^2^*z*^3^*c*_0_ is the Debye length, *r*^2^ = *s*/*D* and *k*^2^ = *r*^2^ +*κ*^2^ and *c*_0_ is the initial concentration. The Laplace component of the thermovoltage is21
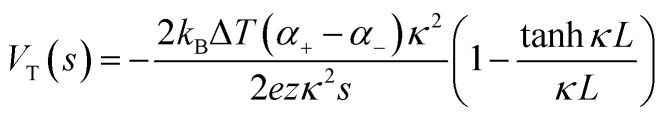


To obtain the time evolution of the neutral concentration, charge density and thermovoltage we have to Laplace transform the above equations, which can be done in the limit of long times. In particular, the thermovoltage takes the expression22
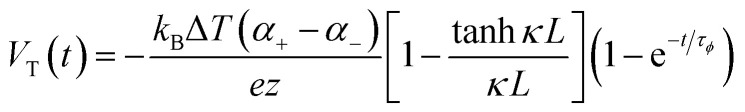
with23
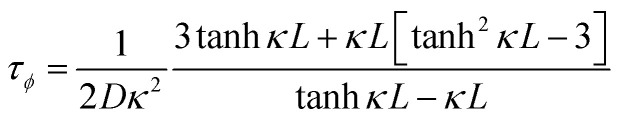
the time scale associated with the thermovoltage evolution. *τ*_*ϕ*_ depends on *κL* and it is proportional to the Debye time 1/*Dκ*^2^. For very thin diffuse layers *κL* → ∞ (the Debye length much smaller than the channel length), *τ*_*ϕ*_ = 1/*Dκ*^2^ is equal to the Debye time. In ref. 120 they find the same expression, eqn (22), once we take the limit *t* → ∞ (stationary state).

A similar treatment can be found in the work of Sehnem, A. L. *et al.*^122^ They study the time evolution of the thermopower when the temperature gradient is established and gradually changed. They also use the Laplace transform to find the solution of the Nernst–Planck, Poisson and heat equations. As in the previous case, the lateral dimensions *d* is small compared to 2*L*, the distance between the electrodes, thus the system is considered isothermal in the lateral direction. The temperature difference is assumed to be

24 Δ*T* = Δ*T*_∞_*h*[1 − e^−*t*/*τ*_ap_^]

where Δ*T*_∞_ is the late-time Δ*T* and *τ*_ap_ is taken to be 43 s, a characteristic time scale.^122^ When the temperature of the hot electrode increases stepwise, the electrolyte relaxes thermally with a time scale *τ*_T_ = 4*L*^2^/π*a*^2^, *a* being the diffusivity. In the experiment they perform, 2*L* = 5 mm, *a* = 1.4 × 10^−7^ m^2^ s^−1^, yielding *τ*_T_ = 18 s. Since *τ*_ap_ ≳ *τ*_T_ they consider a linear temperature profile:25
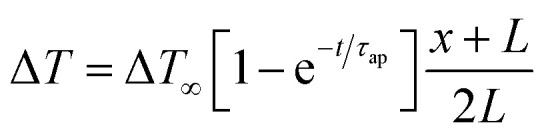
and the ionic current simplifies to26
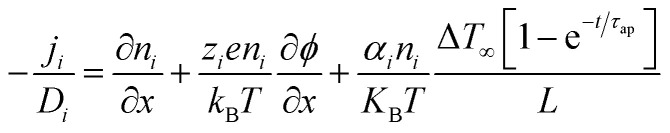


Then, they introduce the parameters *ε* = Δ*T*_∞_/*T*, where Δ*T*_∞_ is the temperature gradient in the final or stationary state, and *n* = *Lκ*, where *L* is the length of the channel and 1/*κ* the Debye length. With the approximations *ε* ≪ 1 (small temperature gradients) and *n* ≫ 1 (in their experiments *n* > 3.5 × 10^6^) they found an analytical solution for the thermopower:27
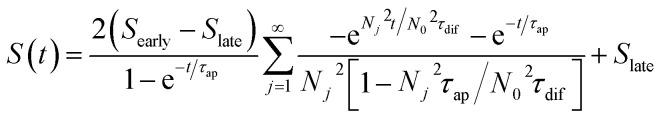
where *S*_early_ is the Seebeck coefficient when the temperature gradient is established, *S*_late_ is the Seebeck coefficient at the stationary state (*t* → ∞), *N*_*j*_ = (*j* − 1/2)π and *τ*_dif_ = 4*L*^2^/π^2^*D*_a_, the ambipolar salt diffusivity,28
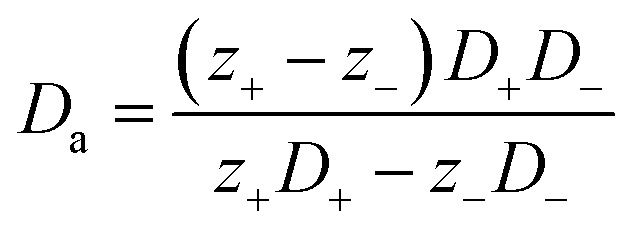


**Fig. 18:**
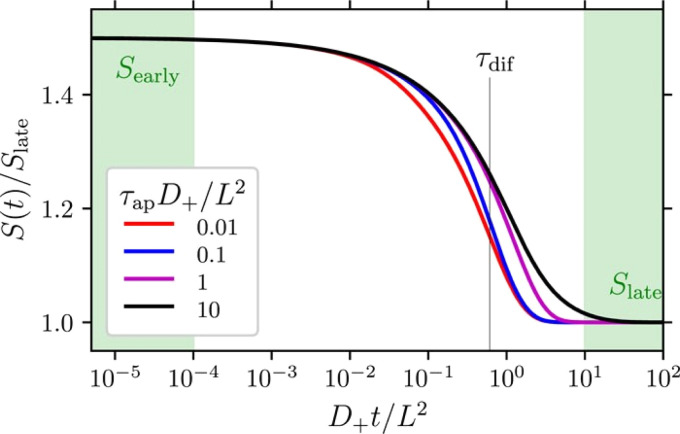
*S*(*t*)/*S*_late_ (eqn (27)) for several values of *τ*_ap_*D*_+_/*L*^2^ [0.01 (red), 0.1 (blue), 1 (magenta), and 10 (black)] with *ξ* ≡ *D*_+_/*D*_−_ = 2, *α*_+_ = 0.5, *α*_−_ = 0.1, *z*_+_ = −*z*_−_ = 1, and max(*j*) = 500 throughout. For these parameters, *S*_early_ = (3/2)*S*_late_.

In Fig. 18 we can see the time evolution of the Seebeck coefficient with the parameters given in the caption. The Seebeck coefficient remains practically constant until *t* ∼ 10^−2^*τ*_dif_, then it decreases to the stationary value at a time along the diffusion time. In the article, Sehnem A. L. *et al.*^122^ present also experimental results using different electrolytes. They obtain from the fitting with the theory the ambipolar diffusion coefficient and the time scale to which thermovoltage relax to equilibrium (steady state).

#### 7.2.2 Two-dimensional case

**Fig. 19:**
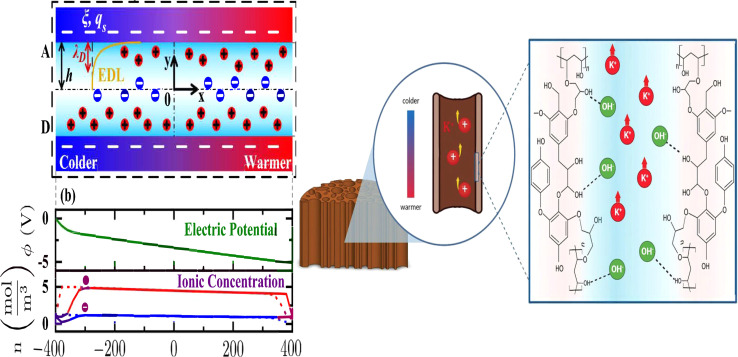
Left panel: The channel drawn in Fig. 14 (length 2*L* along *x*) has now a lateral dimension of width 2*h* (along the *y*-axis) confining the electrolyte. The positive ions moving towards the wall create charges in the Electric Double Layer (EDL). Right panel: cylindrical channel from lignin with an electrolyte and K^+^ and OH^−^ ions.

Qian *et al.*^120^ and Zhong *et al.*^123^ analyze the two-dimensional case (see Fig. 19, left panel) considering the steady state problem. More recently, Muddasar *et al.*^30^ study the case of cylindrical geometry (capillary). In Fig. 19, left panel, upper part, a two-dimensional (2D) channel has been drawn, with length 2*L* = 800 nm and width *h*. The negative ions trapped in the wall form with the positive moving ions an electron-double-layer (EDL), producing a lateral potential despite the potential difference existent in the channel due to the Seebeck effect. The total charge is zero, thus there will be a smaller concentration of cations in the middle of the channel. Below this figure, the electric potential and the ion concentration along the *x* direction is shown. The right part of the figure represents a lignin capillary, with OH^−^ ions trapped in the walls in this case and a current of positive K^+^ ions along the channels.

The physical problem of a layer and a capillary is equivalent, we will analyze here that of a capillary, which is mathematically a little bit more complex. The starting point is eqn (7), (11), (12) and (13), written in cylindrical coordinates. The problem has no *φ*-dependence, thus *n*_*i*_(*r*, *z*) and *ϕ*(*r*, *z*), but since the temperature gradient is established between the points *z* = −*L* and *z* = *L*, the temperature will depend only on *z*. In the stationary case ∂*n*/∂*t* = 0, thus ∇·*j* = 0, *i.e.*29
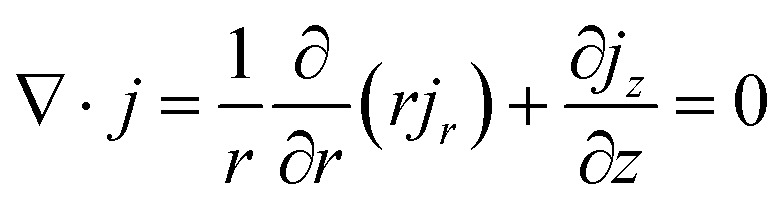


But the radial component of the current must be zero, the particle flux will be established along *z*. Thus, on one hand we have30
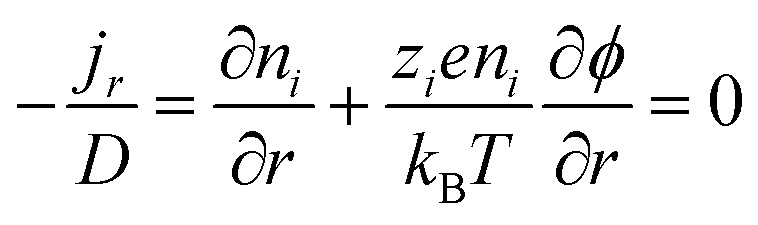


On the other hand,31
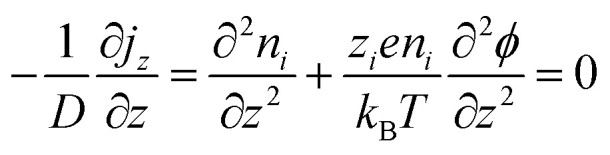


Let us call *ψ* = *z*_*i*_*eϕ*/*k*_B_*T*, the two equations can be rewritten as32
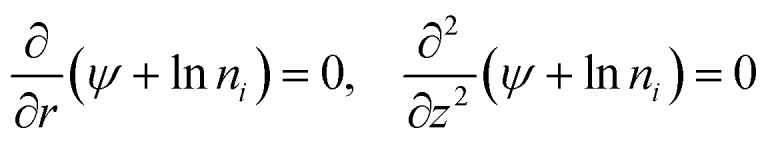


From the solution of the two equations, *ψ* + ln *n*_*i*_ = *cz*, *c* being a constant. The constant *c* must be zero, otherwise the concentration would increase exponentially along *z*. Thus

33 *n*_*i*_(*r*, *z*) = *n*_*i*,0_e^−*ψ*^

## 8. Future direction for practical applications

Despite significant advancements in i-TE materials and devices over the past decade, the efficiency of i-TE devices still does not align with the performance of the materials used. High electrical and thermal contact resistances are primary reasons for the lower conversion efficiency at the device level. Enhancing both the flexibility and thermoelectric properties of i-TE materials to construct energy storage devices remains one of the biggest challenges.

**Fig. 20:**
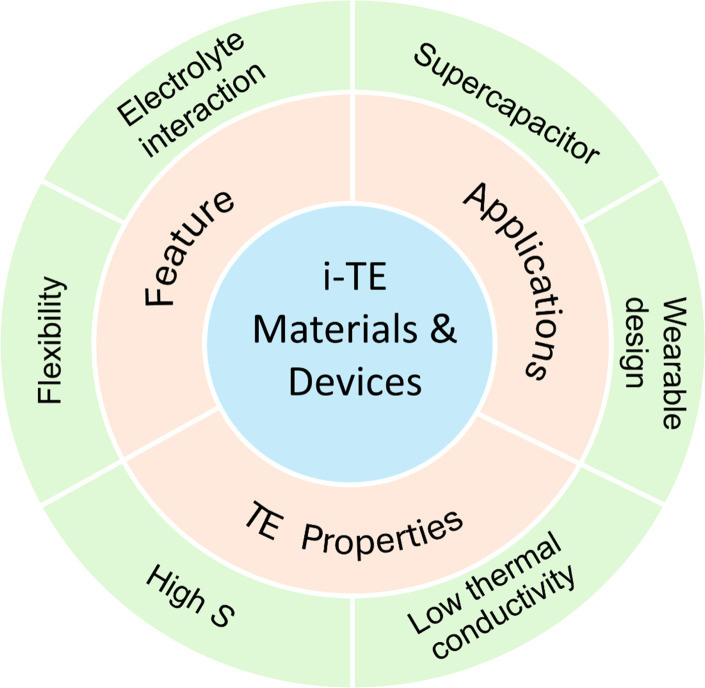
Future directions and outlooks for i-TE materials and devices.

Extensive studies on device manufacturing and electrical and thermal contacts are required to improve the conversion efficiency of energy conversion and storage devices. Additionally, improved fabrication methods could enhance the performance and robustness of thermoelectric devices. The ultimate goal of improving the performance of i-TE materials is their practical application. We highlight the outlook for future high-performance i-TE materials and devices, as summarized in Fig. 20.

### 8.1 Reducing thermal conductivity

Research on i-TEs has primarily focused on enhancing ionic thermopower and ionic conductivity for efficient heat conversion. While i-TEs inherently exhibit low thermal conductivity, reducing it further by an order of magnitude could significantly improve device performance.

### 8.2 Electrolyte interaction

The thermopower of thermally diffusive i-TEs is significantly higher than that of thermogalvanic i-TEs, suggesting promising and diverse potential applications. However, a major challenge is that ions struggle to pass through electrodes unless utilizing the ion-electron thermoelectric synergy (IETS) effect to directly power an external connection. This limitation currently restricts many of these materials to functioning as supercapacitors, with intermittent charging and discharging reducing current density and conversion efficiency. Addressing this challenge is urgent, and potential solutions could involve leveraging the combined effects of electrolyte interactions.

### 8.3 Electrode interaction and design

Future exploration of i-TEs should consider the critical impact of electrodes. Innovative methods to tackle the issue of intermittent charging and discharging are anticipated. Since i-TEs, when used as electrolytes, inherently require electrodes, the choice of electrodes is crucial for influencing thermoelectric properties. Enhancing these properties can be further achieved through thoughtful electrode design that incorporates the thermoelectric effect.^124^

### 8.4 Developing novel designs for i-TE devices

Despite advancements in i-TE device development, challenges persist in waste heat harvesting, efficient heat transfer, scalability, and durability. Utilizing ultrathin, flexible films with a modulus matching thermoelectric materials as substrates could facilitate the fabrication of high-performance TE devices capable of withstanding repeated bending. This approach may address critical issues such as high interfacial resistance between electrodes and TE legs, and overall mechanical durability in flexible devices.

### 8.5 Commercialization of flexible i-TE devices

The compatibility of i-TE materials with polymer substrates makes 3D printing a feasible method for fabricating flexible i-TE devices. However, the field is still in its infancy, requiring further research to develop robust techniques for ink preparation and 3D printing that minimize morphological defects and maximize mechanical stability.^48^

### 8.6 Advanced 3D textiles

One promising research avenue is the development of revolutionary i-TE devices based on 3D textiles. The fabrication methods for 3D textile devices need improvement due to their superior breathability and flexibility. Computational methods, such as finite element numerical simulation, are expected to assist in the design of textile-based devices. Additionally, establishing evaluation standards is crucial for future commercial applications.

### 8.7 Bio-based and waste recycling in i-TE

The current low thermoelectric performance and device efficiency necessitate the exploration of alternative resources. These include recycled carbon fiber, the development of biodegradable thermoelectric papers, and the extraction of useful elements from waste or recycled materials, such as spent modules.

### 8.8 Multifunctional wearable design

Despite advancements in preventing device failure, the limited energy conversion efficiency of flexible i-TEGs remains a challenge. To maximize energy output for commercial applications, exploring multifunctional wearable devices integrating pyroelectric, triboelectric, piezoelectric, magnetoelectric, and photovoltaic functionalities is crucial.^125^

## 9. Conclusion and perspective

In recent years, ionic thermoelectric materials (i-TEs) have emerged as a promising technology for waste heat harvesting. Unlike traditional electronic thermoelectric (e-TE) materials, i-TEs boast exceptionally high ionic thermopower, making them ideal for capturing and converting low-grade thermal energy. Additionally, i-TEs offer advantages in terms of flexibility, cost-effectiveness, and environmental friendliness. In this review, a unique feature of i-TE devices is their ability to function as both thermoelectric generators and thermal-powered capacitors. This review comprehensively allows them to generate electricity even from fluctuating heat sources. However, due to limitations in ion transport across electrodes, i-TE materials cannot be directly used in conventional thermoelectric generators (TEGs). Instead, i-TEs find application in ionic thermoelectric supercapacitors (ITESCs), which can harvest intermittent heat and exhibit transient voltage behavior. Thermogalvanic cells (TGC) represent another promising avenue for waste heat recovery using i-TE materials. These cells utilize an electrode–electrolyte pairing that maximizes reaction entropy and minimizes internal resistance, enabling continuous current flow. With their potential for cost-effective waste heat cogeneration, TGC hold significant promise for future applications, particularly where solid-state TEGs are economically impractical. The ferricyanide/ferrocyanide couple exemplifies a common choice for thermogalvanic cell electrodes due to its clean and reversible nature. Recent advancements further suggest that the performance of thermogalvanic generators can be significantly improved. This review also highlights the synergistic effect between the thermodiffusion phenomenon and electrode redox reactions, paving the way for the development of high-performance, thermochargeable devices suitable for commercialization. However, the theoretical models presented in this review require further refinement to accurately capture the complexities of ion diffusion, migration, and conduction within i-TE materials.

Several key challenges remain in optimizing i-TE material performance.

• The discovery of novel n-type and p-type i-TE systems, finding a balance between thermoelectric and mechanical properties (flexibility, self-healing, *etc.*), and enhancing material stability. Notably, improving ionic conductivity (*σ*) is crucial for optimizing the thermoelectric behavior of i-TE materials.

• Furthermore, the integration of i-TE materials with other advanced TE components offers exciting possibilities for unlocking their full performance potential. While aqueous electrolytes offer an appealing approach, their limitations in heat and charge transport necessitate further exploration. Molten salt-based alternatives, while offering some improvement, may not justify the increased cost and complexity. Beyond their potential as capacitors, generators, and sensors, the high-efficiency operation of i-TE devices remains paramount.

• Future advancements will require a deeper understanding of high-performance devices, large-scale feasibility, and cost-effective material innovation. This includes exploring novel battery architectures, tailoring materials for specific applications, and developing biocompatible materials for wearable electronics. Additionally, minimizing contact resistance and optimizing electrode materials through targeted research efforts are crucial for unlocking the full potential of i-TE technology.

This review provides a comprehensive survey of advanced i-TE materials, equipping researchers to explore next-generation waste heat recovery technologies that contribute to a sustainable future. As the field of ionic thermoelectrics continues to emerge, this review serves as a valuable handbook, offering a foundational understanding of the current state-of-the-art and propelling advancements in low grade energy harvesting technologies.

## Data availability

No primary research results, no new data were generated or analysed as part of this review.

## Author contributions

Nazish Jabeen: writing – original draft preparation, visualization, review & editing. Muhammad Muddasar, Nicolás Menéndez, & Mohammad Ali Nasiri: writing – original draft preparation. Clara M Gómez & Maurice N Collins: visualization, review & editing. Rafael Muñoz-Espí & Andrés Cantarero: review & editing. Mario Culebras: writing – original draft preparation, supervision, review & editing, funding acquisition.

## Conflicts of interest

The authors declare that there is no conflict of interest.
